# Mesenchymal stromal cells (MSC) from JAK2^+^ myeloproliferative neoplasms differ from normal MSC and contribute to the maintenance of neoplastic hematopoiesis

**DOI:** 10.1371/journal.pone.0182470

**Published:** 2017-08-10

**Authors:** Teresa L. Ramos, Luis Ignacio Sánchez-Abarca, Beatriz Rosón-Burgo, Alba Redondo, Ana Rico, Silvia Preciado, Rebeca Ortega, Concepción Rodríguez, Sandra Muntión, Ángel Hernández-Hernández, Javier De Las Rivas, Marcos González, José Ramón González Porras, Consuelo del Cañizo, Fermín Sánchez-Guijo

**Affiliations:** 1 Universidad de Salamanca-IBSAL-Hospital Universitario, Servicio de Hematología, Salamanca, Spain; 2 Centro en Red de Medicina Regenerativa y Terapia Celular de Castilla y León, Salamanca, Spain; 3 Centro de Investigación del Cáncer, Universidad de Salamanca y Consejo Superior de Investigaciones Científicas (IBMCC, USAL/CSIC), Salamanca, Spain; 4 Departamento de Bioquímica y Biología Molecular, Universidad de Salamanca, Salamanca, Spain; 5 Centro de Investigación Biomédica en Red de Cáncer (CIBERONC), Madrid, Spain; Emory University, UNITED STATES

## Abstract

There is evidence of continuous bidirectional cross-talk between malignant cells and bone marrow-derived mesenchymal stromal cells (BM-MSC), which favors the emergence and progression of myeloproliferative neoplastic (MPN) diseases. In the current work we have compared the function and gene expression profile of BM-MSC from healthy donors (HD-MSC) and patients with MPN (JAK2V617F), showing no differences in the morphology, proliferation and differentiation capacity between both groups. However, BM-MSC from MPN expressed higher mean fluorescence intensity (MIF) of CD73, CD44 and CD90, whereas CD105 was lower when compared to controls. Gene expression profile of BM-MSC showed a total of 169 genes that were differentially expressed in BM-MSC from MPN patients compared to HD-MSC. In addition, we studied the ability of BM-MSC to support the growth and survival of hematopoietic stem/progenitor cells (HSPC), showing a significant increase in the number of CFU-GM colonies when MPN-HSPC were co-cultured with MPN-MSC. Furthermore, MPN-MSC showed alteration in the expression of genes associated to the maintenance of hematopoiesis, with an overexpression of SPP1 and NF-kB, and a downregulation of ANGPT1 and THPO. Our results suggest that BM-MSC from JAK2^+^ patients differ from their normal counterparts and favor the maintenance of malignant clonal hematopoietic cells.

## Introduction

Myeloproliferative neoplasms (MPN) are a group of clonal hematological disorders, arising from hematopoietic stem/progenitor cells (HSPC) harboring genetic defects that promote abnormal proliferation and expansion of mature myeloid cells. According to the 2008 classification of the world health organization (WHO), the classical entities polycythemia vera (PV), essential thrombocythemia (ET), and primary myelofibrosis (PMF)[2, 2] are included among the Philadelphia-negative myeloproliferative disorders. The uniquely acquired somatic JAK2V617F mutation is a clonal mutation that can be detected in the HSPC as well as in mature hematopoietic cells[3, 4]. JAK2V617F mutation is estimated to be present in >95% of PV and 50% of ET and PMF patients[1].

Emerging insight regarding the crosstalk between leukemic cells and their microenvironment supports the notion that changes in the marrow stromal niche influences the proliferation, survival and selection of malignant cells[5]. Bone marrow (BM) is a three-dimensional dynamic structure defined by a complex network of extracellular matrix (ECM) proteins and non-hematopoietic cells, responsible for supporting the growth, maturation and maintenance of HSPC[6]. Mesenchymal stromal cells (MSC) are multipotent progenitor cells, and important components of the BM niche. They are a repository of cells that participates in bone development, maintenance, and remodeling. MSC also are important in the regulation of HSPC through the interaction with other stromal cells, diffusible environment factors, and the release of ECM components[7, 8]. Conflicting results have been published about the role of BM-MSC in the pathogenesis of MPN[9]. Apart from the absence of the somatic mutation JAK2V617F in the BM-MSC of patients, little is known about the characterization and biological behavior of these cells[10]. Some studies reported genetic and functional aberrations of BM-MSC in MPN, which were followed by a decrease in proliferation rated and osteogenic capacities[11]. Others showed that MSC from PMF patients exhibited a persistent increase of their osteogenic abilities[12].

In this study we analyzed the behavior of BM-MSC from MPN patients (PV and ET) with the mutation in JAK2V617F. We excluded PMF patients due to the problems to obtain a representative BM aspiration product. We initially characterized the biological function and gene expression profile changes in BM-MSC from patients when compared to BM-MSC of healthy donors (HD). Then, we established co-cultures between MSC cell lines (HTERT and HS5) and the MPN cell line, to study if the leukemic cells were able to modify the genes related to hematopoietic support.

## Materials and Methods

### Samples and ethical statements

Bone marrow (BM) aspirates from 37 healthy donors (HD) and 33 newly diagnosed MPN patients. Median age of control samples (HD-BM) was 49 (range 31–73), 23 male and 14 female. Patient´s characteristics are summarized in Table 1.

**Table 1:** Clinical characterisitics of MPN patients.

Subject	Gender	Age (y)	Hb (g/dL)	Platelets 10^3^/μL	WBC 10^3^/μL	%JAK2^V617F^
**PV1**	F	46	16.9	775	11.1	17%
**PV2**	M	53	13.9	262	4.01	49%
**PV3**	F	52	17.3	649	8.11	33%
**PV4**	M	74	15.7	233	4.19	40%
**PV5**	F	72	16.4	454	14.13	90%
**PV6**	M	66	16.9	1041	17.6	46%
**PV7**	M	67	17.3	485	12.9	33%
**PV8**	F	69	16.7	356	5.87	33%
**PV9**	M	71	18.4	597	8.26	23%
**PV10**	F	70	16.8	387	17.3	75%
**ET1**	F	40	14.3	415	9.2	24%
**ET2**	F	51	14.1	596	9.17	16%
**ET3**	F	70	17.6	567	8.67	54%
**ET4**	M	52	13.5	567	8.96	11%
**ET5**	M	74	15.7	180	6.08	35%
**ET6**	F	37	14.7	484	6.56	21%
**ET7**	M	65	14.5	483	7.63	20%
**ET8**	M	69	14.9	293	6.79	32%
**ET9**	F	51	13.5	644	7.41	13%
**ET10**	M	72	11.8	472	7.98	48%
**ET11**	F	74	14.3	636	5.83	22%
**ET12**	M	73	12.3	1167	13.7	25%
**ET13**	F	45	15.5	648	10.5	12%
**ET14**	M	73	11.3	656	9.73	60%
**ET15**	F	56	16.1	856	8.5	20%
**ET16**	M	62	17	697	10.2	10%
**ET17**	M	31	16.6	168	3.78	30%
**ET18**	F	68	14.7	571	6.92	18%
**ET19**	M	74	15.8	387	11.6	16%
**ET20**	M	70	14.1	224	3.8	22%
**ET21**	F	67	15.8	1027	11.1	24%
**ET22**	F	50	15.3	517	7.78	15%
**ET23**	M	47	14	634	5.22	44%

F- Female, M- Male, PV–Polycythemia Vera, ET- Essential thrombocythemia, Hb–Hemoglobin, WBC- withe blood cells. %JAK2V617F –represents the percentage of hematopoietic cells with the mutation in the bone marrow or in peripheral blood.

### Ethics

The study was conducted in accordance to ethical standards and principles expressed in the Declaration of Helsinki. Informed consent was obtained from all donors and patients included in the study, and approved by the local Ethics Committee of the Hospital Universitario de Salamanca (Committee´s name) with the reference number 2014/06/86.

### Cell lines

UKE-1 and SET cell line derived from patients diagnosed with MPN, homozygous for JAK2V617F mutation, was used in the experiment. This MPN cell line was kindly donated by Prof. António Almeida[13]. The stromal cell lines used were: hMSC-TERT[14], which was a generous gift from Dr. Dario Campana and the cell line HS-5 purchased from ATCC (ATCC CRL11882). The cell lines were cultured as previously reported[13, 15].

### Isolation and culture of BM-MSC from MPN patients and HD

Bone marrow mononuclear cells (BM-MNC) were isolated and cultured in standard culture medium as previously described[16]. In each passage MSC were cultured until approximately 70–80% confluence was reached, cells were detached and re-seeded for culture expansion at a density of 5000 cells/cm^2^. In the expansion cycle, cells counts were performed after Trypan Blue (Gibco, InvitrogenTM) staining on a Neubauer chamber. Population doublings (PD) were calculated for each group using the following equation: PD = log10(N)/log10(2), where N is the number of cells harvested at the end of the culture per number of seeded cells[17, 18]. Isolated BM-MSC were characterized fulfilling the minimal criteria established by the International Society for Cellular Therapy (ISCT)[19].

For the immunophenotypic characterization of BM-MSC were performed according with previously established protocols[15, 16, 20]. Adherent cells at passage 3 were harvested and incubated using the following conjugated monoclonal antibody combinations: CD34-FITC, CD73-PE, CD45-PerCPCy5.5, CD105-APC/ CD44-FITC, CD14-PE, CD19-PerCPCy5.5/ CD90-FITC, CD166-PE, HLA-DR-PerCPCy5.5 (all from Becton Dickinson–BioSciences, except CD105 that was purchased from R&D systems). To control background fluorescence, unstained MSC were also acquired and used as negative control. Expression of individual markers was recorded, both as a percentage of positive cells and as mean fluorescence intensity (MIF) after subtracting the baseline auto-fluorescence levels observed for MSC.

Data were analyzed using the Infinicyt program (Cytognos, Salamanca, Spain). In order to compare the expression level of each antigen the mean channel fluorescence was calculated subtracting the MSC without staining. For *in vitro* adipogenic and osteogenic differentiation was performed as previously reported[20].

### RT-PCR determination

Total RNA was extracted from BM-MSC and BM-MNC with Trizol (Invitrogen) and subsequent reverse transcription was carried out using the High Capacity kit (Applied Biosystems, Foster City, CA, USA). Gene expression (S1 Table) was quantified by using commercial TaqMan Gene Expression Assays and the Step One Plus Real-Time PCR System (Applied Biosystems). Relative quantification was calculated using the 2−^ΔCt^ values where: ΔCt = Ct_Gene_—Ct_GAPDH_. Panel of genes used in RT-PCR assay in additional information.

### Allelic discrimination assay for JAK2V617F in MSC

Twenty nanograms of genomic DNA from 9 samples of MSC from patients carrying the JAK2V617F in their hematopoietic cells were amplified using a TaqMan SNP genotyping assay (Thermo Fisher Scientific). This assay is based on the simultaneous use of two specific fluorescent TaqMan probes (6FAM for V617F and VIC for wild-type) to differentiate the amplification of each allele. DNA from a patient homozygous for the mutation (100%) was used as positive control.

### Gene expression profiling using microarrays: Determination and data analyses

After assessment of RNA integrity (Agilent 2100 Bioanalyzer, Agilent), samples were hybridized in the platform Gene Chip Human Gene 1.0 ST array system (Affymetrix, Santa Clara, CA), following manufacturer’s instructions. The microarray expression dataset is fully available from the GEO repository (https://www.ncbi.nlm.nih.gov/geo/) under the identifier: GSE87806. Data analyses were performed in the R/Bioconductor statistical environment. Microarrays were pre-processed and normalized with the RMA (Robust Multi-Array Average) algorithm. The algorithm LIMMA was used for differential expression analysis. A second algorithm SAM (Significance Analysis of Microarrays) was used to corroborate and validate the results obtained with LIMMA. The genes selected as significant passed a cut threshold of FDR < 0.05 (Fold Discovery Rate). More details about the way these methods were applied are provided in the Supporting Information. Top significant differentially expressed genes derived from the LIMMA method, were submitted to functional enrichment analysis using the bioinformatics tool, DAVID Bioinformatics Resources 6.7 (http://david.abcc.ncifcrf.gov/)[21, 22]. The gene sets analyzed for functional enrichment were the most significant from List I (up-regulated genes with FDR<0.03) and List II (up-regulated genes with FDR<0.02).

### Apoptosis and cell cycle assays

For apoptosis assessment, the cells from the different experimental condition were stained with Annexin V-PE using the PE annexin V apoptosis detection kit (BD) following the manufacturer´s instructions. For cell cycle, the cells were stained with propidium iodide (PI), using the kit cycle tests (BD), according to manufacturer’s instructions. The samples were acquired in a FACSCalibur flow cytometer and analyzed as previously described[23].

### Immunobloting

Proteins cell extracts and western-blot was performed as reported previously[24] Primary antibodies used were MYADM (kindly provided from Prof. Miguel A. Alonso[25]), NF-Kß, Ang-1 (both from Santa Cruz) GAPDH and Tubulin (Cell Signaling).

### Immunofluorescence

For immunofluorescence experiments, BM-MSC were fixed with 4% paraformaldehyde and was performed as previously described[26].

### Isolation of hematopoietic progenitor cells

After density gradient separation, CD34^+^ cells were purified from BM-MNC or from leukapheresis samples from 4 HD (with a male/female ratio of 3/1, median age 26.5 years; range 22–46 years) by immunomagnetic cell separation (Miltenyi Biotec, Bergisch-Gladbach, Germany) as previously described[20].

### Hematopoietic support assessment

BM-MSC derived from JAK2V617F patients and HD were seeded at a density of 1X10^5^cells/well in 24-well plates and cultured overnight. Purified CD34^+^cells (1x10^5^-2x10^5^) from BM of MPN patients and HD were co-cultured using a *transwell* membrane of 0.04μm (Costar, Corning NY, USA). After 48 hours of co-culture, HSPC were recovered and 5x10^3^ cells were seeded into methylcellulose MACS Media with Stem Cell Factor, GM-CSF, G-CSF, IL-3 and IL-6 (MethoCult H4534-Stem Cell Technologies, Vancouver, Canada) to quantify only the progenitor cell colony-forming unit–granulocyte/macrophage (CFU-GM), according to the manufacturer’s instructions. After 14 days, CFU were enumerated and classified by morphology as previously described[27].

### Long-term bone marrow cultures (LTBMC)

BM-MSC from MPN patients and HD were maintained in culture until confluence was achieved. The expansion medium was changed for LTBMC medium according to the methods of Gartner and Kaplan with slight modifications and as previously described[20, 28]. During 5 weeks, cells were weekly harvested and plated in methylcellulose medium. After 14 days, colony numbers were scored using an inverted microscope. Results were expressed as number of CFU per 10^5^ cells seeded and the total number of CFU present in the culture.

### Co-culture system

Human mesenchymal cell line hTERT and HS5 were plated at concentration of 10^5^ cells/well in a 6-well plate (Costar, Germany) overnight. Then the cells were incubated with the medium from UKE-1 cells (that were previously centrifuged and filtered to eliminate the cells) or with 4x10^5^ cells/mL of UKE-1, SET-2 and CD34^+^ cells (obtained from leukapheresis) in a *transwell* system constituted by a polycarbonate membrane (3.0μm pore size, Corning Incorporated, Costar). Cultures were maintained in a 5% CO_2_, humidified atmosphere at 37°C for 72 hours. After the time of culture MSC were recovered and processed for total RNA extraction.

### Statistical analysis

Statistical analysis was performed using SPSS software version 21 (Chicago, IL, USA) and GraphPad Prims version 5.00 for Windows (GraphPad Software). The values reported in the figures are given as median with the interquartile range or with mean ± standard error of the mean (SEM). Differences between populations were calculated using the Mann-Whitney tests with Bonferroni corrections. A p-value <0.05 was considered to be statically significant. The outliers were analyzed using the Grubbs´s test (Graph Pad).

## Results

### Characterization of BM-MSC derived from MPN-JAK2^+^ patients

**Fig 1:**
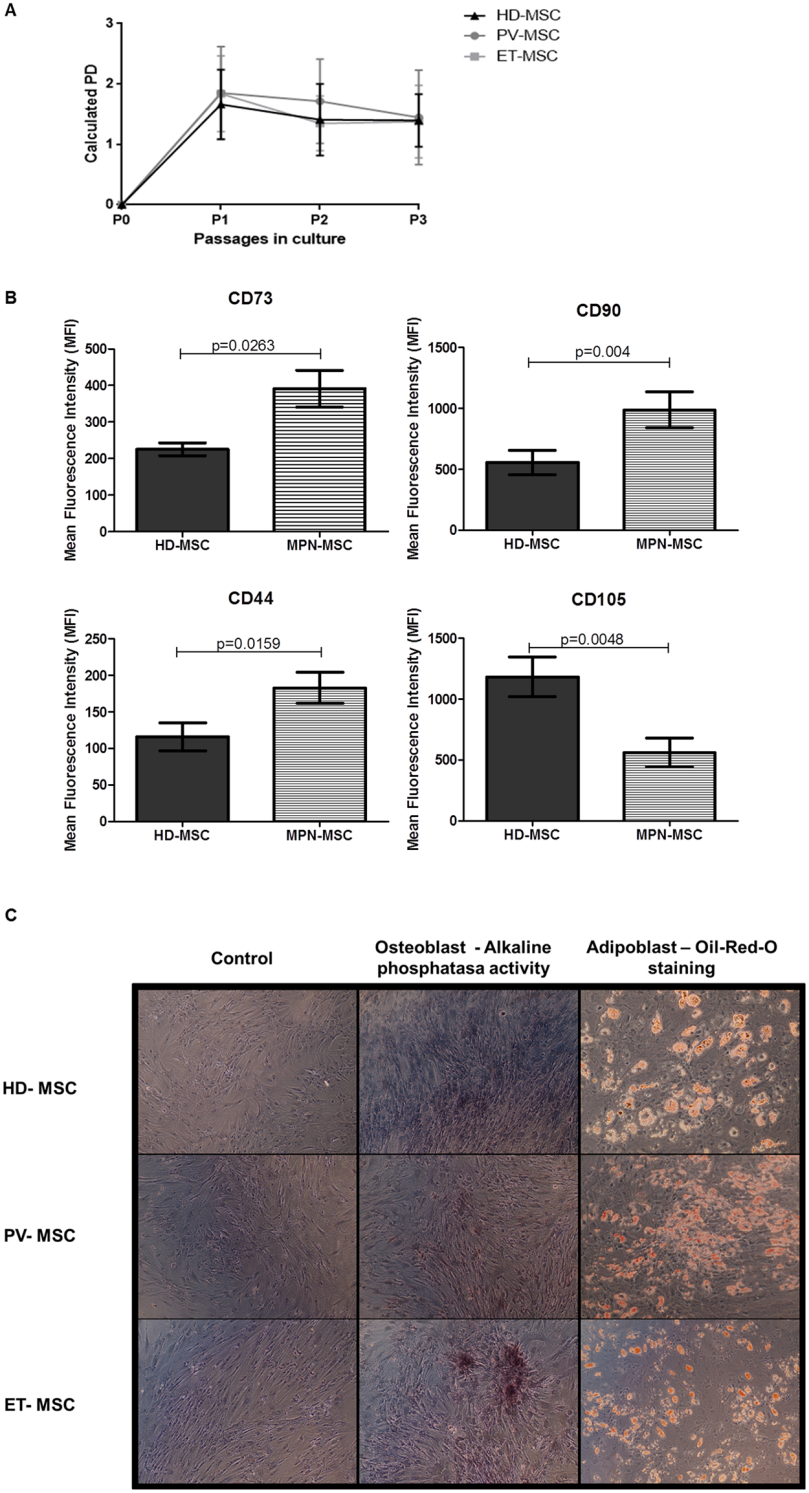
Characterization of BM-MSC from JAK2V617F patients and healthy donors. (A) The number of population doublings (PD) in each passage was calculated using the following equation: PD = log10(N)/log10(2), where N is the number of cells harvested at the end of the culture per the number of seeded cells, where isolated from HD and JAK2^+^ PV and ET patients BM-MSC (Passage1 to Passage 3). (B) Mean fluorescence intensity of positive surface marker expression of HD-MSC and MPN-MSC; HD-MSC n = 20 and JAK2-MSC n = 30. Values indicate the mean ± SEM. (C) In vitro multilineage differentiation assays performed in HD-, PV- and ET-MSC. Left-handed photos represent the negative controls (no induction medium applied). The photos of the middle show osteogenic differentiation detected by alkaline phosphatase activity. Right-handed photos show adipogenic differentiation detected fat staining with Oil-Red-O (20X).

Patient´s characteristics are summarized in Table 1, MPN diagnosis was established according with 2016 revision to of the World Health Organization classification of myeloid neoplasms and acute leukemia classification[29]. BM-MSC from patients with JAK2 mutation displayed similar morphology compared to HD BM-MSC. Fig 1A shows no differences regarding proliferative capacity (expressed in population doubling) between PV/ET-MSC and HD-MSC.

Immunophenotypic characterization, in accordance with the minimal definition criteria established by the ISCT[19], presented in Fig 1B showed that both HD and MPN-MSC were positive for the typical MSC markers, whereas they were negative for hematopoietic markers or the MHC class II molecules, including HLA-DR. When analyzed the fluorescence intensity of surface antigens, an increased expression of CD73, CD90 and CD44 was observed (Fig 1B) in MPN-MSC compared to HD-MSC. In addition, a lower expression of CD105 was found (p˂0.01). Similar expression pattern were observed between PV-MSC and ET-MSC (S1 Fig).

Osteoblastic and adipocyte *in vitro* differentiation was achieved with similar efficiency between both groups (Fig 1C). However, 1 out of 9 PV-MSC samples did not differentiate into adipocytes and 2 out of 20 samples of ET-MSC did not differentiated, one into adipocytes and another into osteoblasts. All HD-MSC samples were capable to differentiate into both lineages.

We verified in a set of 9 samples that the JAK2V617F mutation was absent in MSC derived from patients carrying this mutation in their hematopoietic cells.

### Cell cycle and apoptosis of MPN BM-MSC

**Fig 2:**
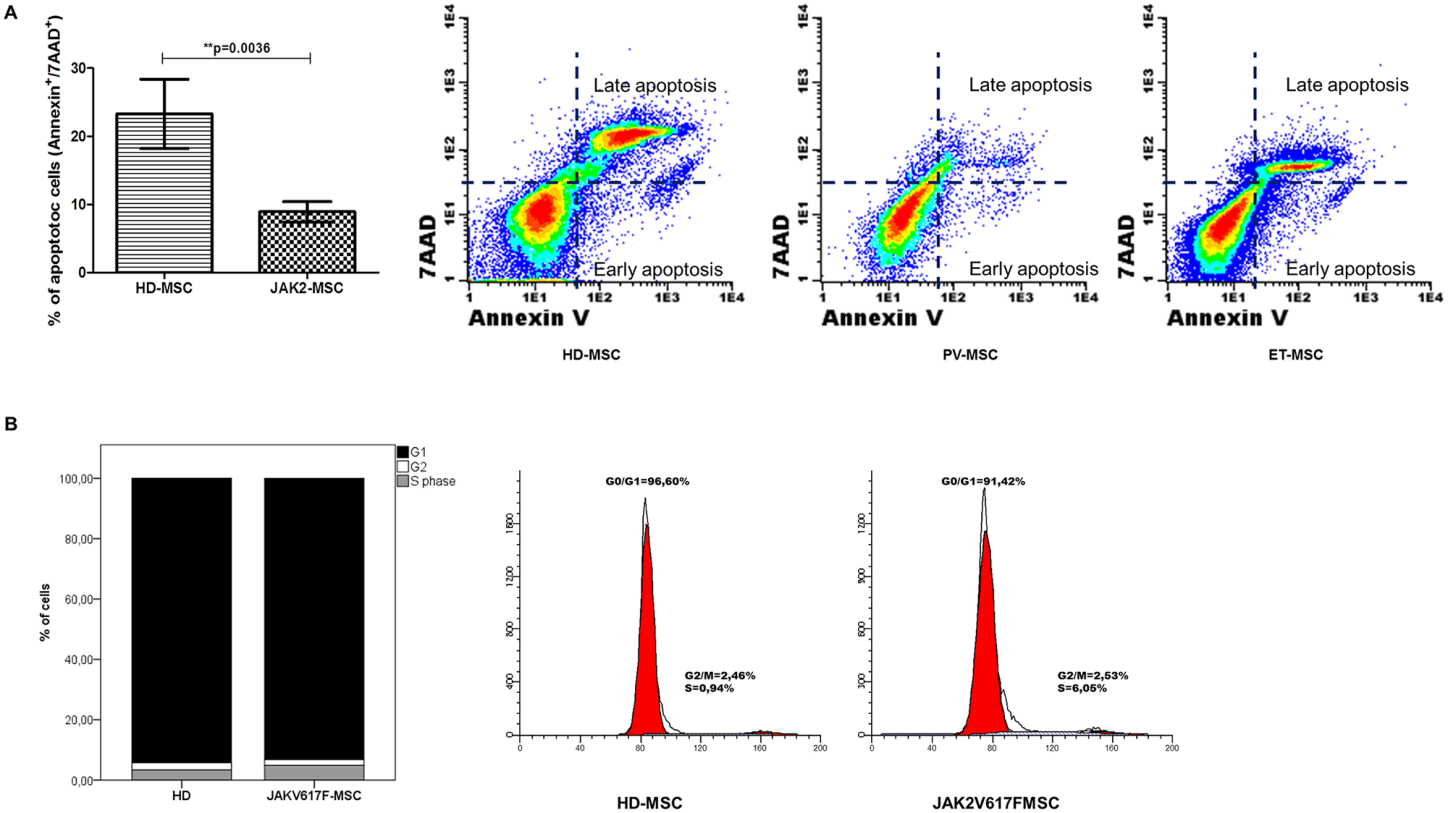
Apoptosis and cell cycle analysis of BM-MSC. (A) Graph bar chart that represents the percentage of apoptotic cells (AnnexinV/7AAD positives), and representative FACS dotplot of annexinV/7AAD staining on BM-MSC.* p˂0.05. (B) Percentage of cells in G1, S and G2/M phase from HD-MSC, PV-MSC and ET-MSC. No differences were observed between groups.

No differences were observed between groups in the cell cycle, the percentage of G0/G1 (HD-MSC = 94.5% (86.9–98.4) n = 10; JAK2-MSC = 95.3% (66.4–97.9) n = 15) and S phase (HD-MSC = 2.4% (0.4–12.93) n = 10; JAK2-MSC = 1.91% (0.24–33) n = 15) (Fig 2A). The percentage of apoptotic cells (annexin^+^/7AAD^+^) was lower (p = 0.0036) in JAK2-MSC compared to controls (HD-MSC = 18.15% (3.8–67) n = 14; JAK2-MSC = 10% (0.1–23) n = 20) (Fig 2B).

### MSC gene expression profile results

To obtain a global view of the differences between HD and MPN cells, the genetic profiles of BM-MSC from 8 JAK2V617F patients (4 PV and 4 ET) and 10 HD were analyzed.

**Fig 3:**
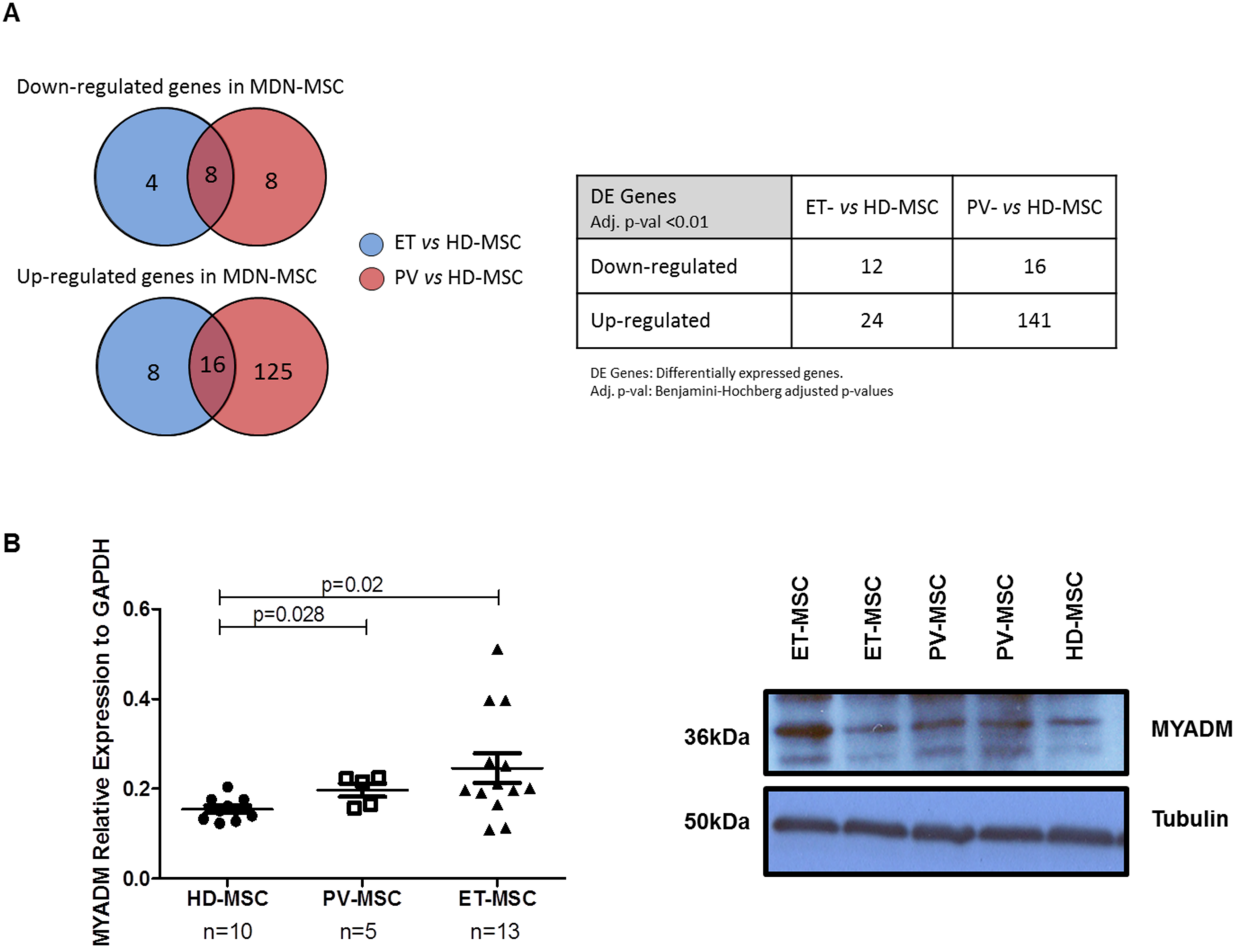
Gene expression profile of BM-MSC from HD and MPN (ET and PV) patients. (A) Represents the number of genes Up and Down regulated in each group when compared against HD group. (B) Gene and protein expression of MYADM gene tested in BM-MSC from MPN patients and HD. Results were normalized with the housekeeping gene GAPDH. Results are represented by the median and the interquartile range. HeLa cells were used as positive control.

Compared to HD, a total of 169 genes were differentially expressed in BM-MSC from MPN patients (S2–S4 Tables). Overall, 125 genes were up-regulated and 8 genes were down-regulated in PV BM-MSC. In ET BM-MSC 8 genes were differentially up-regulated and 4 genes down-regulated (Supporting information). Twenty-four differentially-expressed genes were common in PV and ET-MSC (Fig 3A). Myeloid-associated differentiation marker (MYADM) gene and myeloid leukemia factor 2 (MLF) gene were over-expressed in both PV and ET cases, when independent comparisons to HD-MSC were performed. MYADM expression it was found to be selectively expressed in human myeloid cells (from HD), and also significantly increased in MPN cell lines[30]. In order to confirm the overexpression of this gene observed in array, RT-PCR and WB were performed in the BM-MSC. We observed an over-expression of MYADM (RNAm and protein) in PV and ET respect to healthy donors (Fig 3B).

### Differential biological processes between BM-MSC from MPN patients and healthy control

The differential signal pathways are shown according to the enrichment score value in Table 2. The total genes and representative gene title in respective signal pathways are also shown. The differential signal pathways included endoplasmic reticulum, protein transport, GTPase activity and membrane/ transmembrane region.

**Table 2:** Differential pathways of BM-MSC between MPN-JAK2^+^ (PV and TE) and healthy controls.

***Pathways UP- regulated genes***	**Total genes**	**Enrichment Score**	**Representative gene title**
*Endoplasmatic reticulum*	76	4.3	CYB5R3, SLC36A1, VAPB, MRVI1, ALG3, RCE1, ALG8, C14ORF1, CANT1, TAPBP, ELOVL1, TMEM173, PLOD1, PIGB, RPL10, PSENEN, KDELR1, AGPAT1, KDELR3, PGAP2, KDELR2, PIGU, PIGS, TECR, LPCAT4, UGT2B11, ASPHD1, ORMDL2, LEPREL1, FKBP10, TRAPPC1, EXT2, SLC27A2, SEC61G, CLN6, EXTL3, DERL1, CACNB1, UBE2V1, PPT2, CTSA, TRAM2, STT3A, SHISA5, EMD, PLP2, NPLOC4, ICMT, PORCN, NCSTN, BFAR, C3ORF52, TXNDC11, LASS2, DPM2, TAPBP, BFAR, NRBP1, SLC22A18, C16ORF70, COPZ1, TAGLN2, SLC35A4, NUP214, NECAP1, QSOX1, AP2M1, HIP1, SCAMP2, NRM, KPNA6, VAMP2, FAF1, MGAT5, PEX11B, MPV17
*Protein transport*	75	3.15	SNAP29, RAB7A, DERL1, RAB5C, COPZ1, VPS52, RAB1C, RAB1B, NUP214, TRAM2, NECAP1, KDELR1, SCAMP4, AP2M1, KDELR3, KDELR2, PRAF2, RAB8A, SCAMP2, RABIF, RAB4B, STXBP1, TIMM22, TOM1L2, ARF3, ARF4, KPNA6, RAB15, SEC61G, VPS25 GRPEL1, C16ORF70, CACNB1, CTSA, TRAM2, TOMM34, GDI1, ICMT, YWHAE, YWHAH, YWHAQ, PAX6, FAF1, SLC16A13, SLC36A1, SLC22A18, SLC35A4, GOT2, DIRC2, NDUFS4, ANO10, CYB5B, ATP6V1F, SLC35E1, TRAPPC1, RBP4, SLC39A11, CACNB3, FXYD5, CYB561D2, TRAM2, SLC35B4, SLC4A8, SLC25A44, TCIRG1, ATP5J2, NDUFA2, ATP5F1, SLC16A2, AFM, LASP1, SDHC, TAPBP, MAP1S, VAMP2.
*GTPase activity*	47	2.99	RAB7A, RAB5C, RAB4B, TUBB, GNB1, ARF3, ARF4, RAC1, RRAS, TUBA3D, RHOC, TUBG1, TUBG2, GNG5, NKIRAS2, TUBB3, RHOG, RAB8A, KIF3C, RAB1C, RAB1B, RAB15, GDI1, RABIF, ARHGAP1, CFL1, YWHAQ, ARHGDIA, ACTB, CRYAB, HIST1H2BM, GORASP2, CALM3, PRPS1, CYB5R3, COPZ1, CACNB1, ILK, MSN, AP2M1, TOMM34, MUC1, APOBEC3C, GOLGA7, PLSCR3, DYM, PRKACA
*Membrane/Transmembrane region*	191	2.55	TGOLN2, SLC36A1, RNASEK, NRBP1, SLC22A18, PEAR1, VAPB, CSPG4, RAB1C, VPS52, RAB1B, MPV17, RCE1, FAM119B, C14ORF1, COX5A, SLC35A4, TAPBP, GOT2, OR8K1, ELOVL1, DIRC2, PLOD1, ILK, RRAS, PSENEN, TMEM185B, RNF34, GNG5, SCAMP4, SCAMP2, C1ORF212, TECR, SIRPA, LPCAT4, MARK2, C1ORF85, OR8J1, KIAA0090, TMEM106A, MGAM, RAB15, ASPHD1, NRSN2, TMEM184B, VAMP2, MGAT5, NEU3, FAM171A2, EXT2, SEC61G, FAM171A1, MAVS, EXTL3, DERL1, TMEM214, COPZ1, DAG1, CACNB1, UBE2V1, FXYD5, MANSC1, TRAM2, TMEM127, GORASP2, RAC1, PTPLA, EMD, AP2M1, HIP1, MUC1, RAB8A, PRAF2, ATP5J2, C17ORF101, COX8A, CD276, PPAPDC1A, ICMT, TMEM110, GPR137C, TMEM179B, PORCN, BFAR, EI24, TXNDC11, LASS2, TBXA2R, ENG, PIP4K2C, DCXR, NYNRIN, SLC16A13, CYB5R3, OR10A3, OR5H1, LRRC8A, GPR160, RAB5C, MRVI1, ALG3, TMEM62, ALG8, FAM57A, CANT1, TMEM175, CD97, EFHD2, TMEM173, GOLGA7, NDUFS4, SMAGP, PIGB, NECAP1, RHOC, MSN, TM9SF4, KDELR1, RHOG, COX16, TUBB3, TMEM104, ANO10, AGPAT1, TOMM34, KDELR3, KDELR2, PGAP2, FLOT2, RAB4B, PIGU, STXBP1, PIGS, CYB5B, SLC9A3R1, MYADM, TIMM22, PRKD1, KIAA1161, CLECL1, NRM, SLC35E1, ZDHHC12, UGT2B11, MOSPD3, ORMDL2, MFSD11, C2ORF24, SLC27A2, CLN6, SNAP29, USP30, SLC39A11, SAMM50, CD248, PCDHGC5, ZDHHC18, ITM2C, CYB561D2, STT3A, SHISA4, SLC35B4, SHISA5, PLEKHO1, SLC4A8, HRCT1, SLC25A44, MANBAL, SELPLG, QSOX1, TEX261, GBA, TCIRG1, PTPRB, PLP2, NDUFA2, ATP5F1, TMEM53, AXL, NLGN2, CBARA1, NID2, NCSTN, PEX11B, SLC16A2, C3ORF52, SDHC, PLSCR3, DPM2, CMTM7, VPS25
***Pathways Down- regulated genes***	**Total genes**	**Enrichment Score**	**Representative gene title**
RNA binding/ RNA recognition motif, RNP-1	9	1.46	DDX55, SFRS5, SURF6, ZMAT3, RPS14, SART3, EWSR1, RPS3, SAFB2
structural molecule activity	8	1.32	ISCU, RPL23, COL21A1, RPS14, RPL7L1, COL24A1, COL16A1, RPS3
zinc finger region	8	1.08	ZNF516, ZNF236, ZNF274, KLF9, YY1, PRDM5, ZNF460, ZNF498
extracellular matrix/ biological adhesion	10	1.06	SMOC2, COL24A1, COL16A1, NTN1, COL21A1, IL16, ADAM17, PARD3, TNFRSF10B, GABRB3

### BM-MSC from MPN patients promotes the expansion of leukemic cells

**Fig 4:**
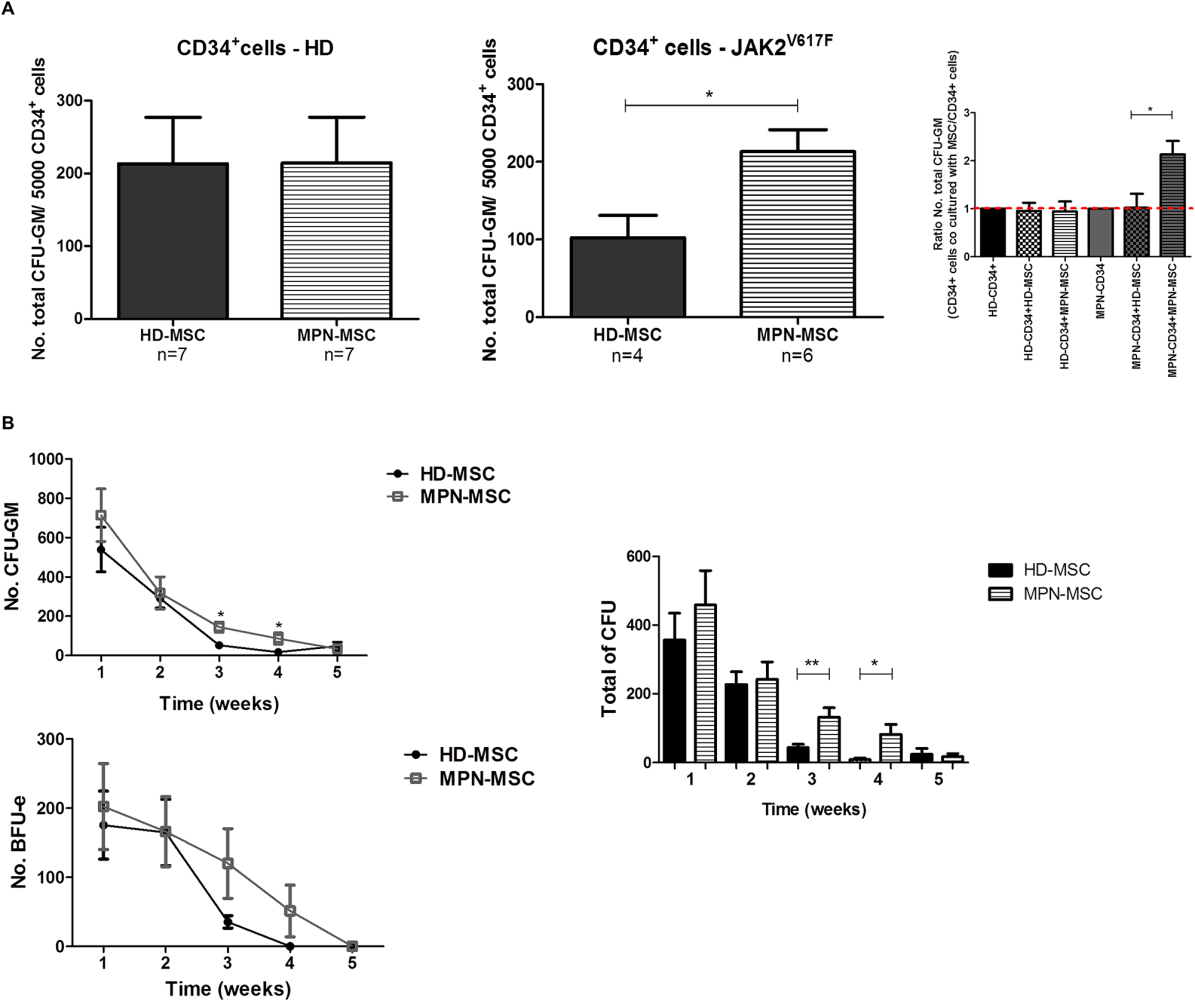
Capacity of MPN-MSC to support hematopoietic progenitor cells. (A) Selective protection of leukemic hematopoiesis by MPN-MSC. Left-handed graphic shows the total colony-forming unit (CFU-GM) from HD-CD34^+^ cells after 48h of culture with HD-MSC (n = 7) and MPN-MSC (n = 7), no differences were observed between groups. Middle-handed shows CFU-GM from JAK2V617F-CD34^+^ cells after culture with HD-MSC (n = 4) and MPN-MSC (n = 6), showing a significant increase when leukemic progenitor cells were cultured with MPN stroma. Right-handed graphic showed the results are expressed as the ratio between CFU-GM obtained with CD34^+^ cells that had been co-cultured with HD-MSC or MPN-MSC and CD34^+^ cells without MSC. The results are showed as absolute CFU-GM per 5000 CD34^+^ cells seeded in methylcellulose, after 14 days in culture. (B) Capacity of MPN-MSC to maintain HD-HPC in LTBMC. Total of CFU-GM from HD-CD34+ cells after 5 weeks in co-culture with HD-MSC (n = 7) and MPN-MSC (n = 7). Total BFU-e. Total number of colonies (CFU), results shown are expressed as the mean of CFU±SEM. * p˂0.05 ** p˂0.01.

As represented in Fig 4A, the total number of CFU was similar when HD-HSPC were cultured on healthy and neoplastic feeder layers. However, a significant increase (p˂0.05) of CFU was observed when CD34^+^ cells from JAK2 patients were cultured with MPN-MSC, showing their capacity in promoting the expansion of functional leukemic progenitors cells. It was not observed differences in the capacity to maintain JAK2-HSPC between BM-MSC from PV and ET patients (S2 Fig).

LTBMC were performed to assess MPN-MSC capacity to support healthy hematopoiesis for 5 weeks. Results showed that BM-MSC from JAK2 patients (n = 7) and from HD (n = 7) exhibited a similar capacity to support normal hematopoiesis during the first 2 weeks. At the 3rd and 4th week, a significant increase of CFU production was observed (week 3—p = 0.021 and week 4—p = 0.0028) when HD-CD34^+^ cells were co-cultured with MPN-feeder layers (Fig 4B). At the fifth week no differences were observed when the HSPC were cultured with both MSC (HD and MPN). For the LTBMC experiments we used 3 BM-MSC from PV patients and 4 samples from ET patients, we observed that ET-MSC showed more capacity to maintain the HD-CD34^+^ cells, without differences between the groups.

### BM-MSC from JAK2V617F patients express different expression pattern of hematopoietic supportive genes

**Fig 5:**
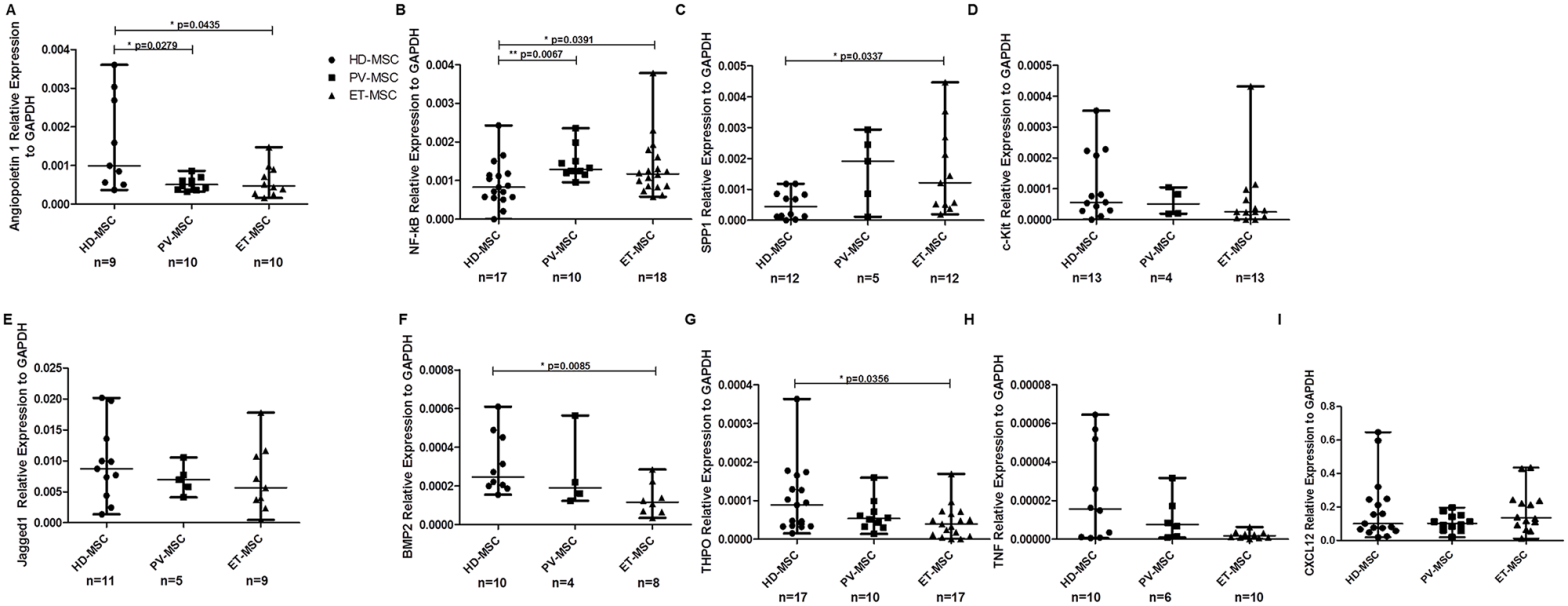
Differential expression of genes related to hematopoiesis in MPN-MSC. RT-PCR was used to determine the expression level of the different genes associated with maintenance of hematopoiesis. GAPDH was used as housekeeping to normalize the results. In the scatter plot graphic are represented by the median and the range.

**Fig 6:**
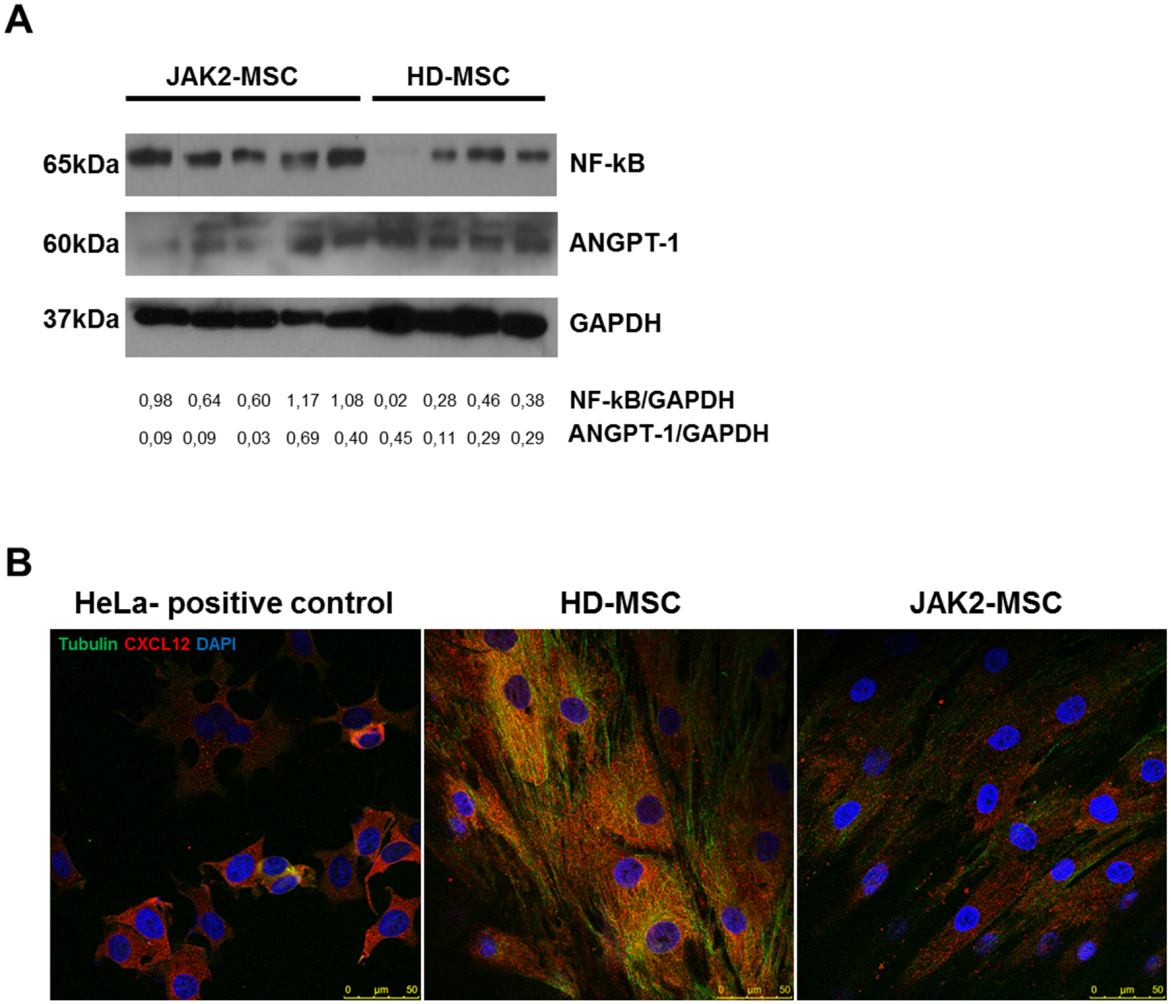
Protein expression of NF-ƙB, ANGPT-1 and CXCL12 in MPN-MSC. (A) Western Blot of NF-ƙB and ANGPT-1 in MPN and HD-MSC. (B) Representative image of CXCL12 expression in the MSC by immunofluorescence. HD-MSC shows more CXCL12 (red) than the positive control (HeLa cells) and the MPN-MSC. In green shows tubulin. Scale: 0–50μm.

Given the changes observed in the capacity of MPN-MSC to support the formation and expansion of healthy and leukemic HPC, quantitative RT-PCR was performed to assess the expression of genes involved in hematopoiesis maintenance. The genes studied were CXCL12, Angiopoetin-1 (ANGPT1), nuclear factor kappa B (NF-kB), Jagged-1 (JAG-1), bone morphogenetic protein 2 (BMP-2) secreted phosphoprotein 1 (SPP1), thrombopoietin (THPO), c-KIT and tumor necrosis factor (TNF) (Fig 5). A downregulation of ANGPT1 (Fig 5A) was observed in ET and PV-MSC. However, an infra-expression of BMP2 and THPO with significant difference was observed in ET-MSC, when compared to HD-MSC. An overexpression of NF-kB was observed in both MPN-MSC (PV and ET) when compared to HD-MSC (Fig 5B). Regarding the expression of SPP1, it was observed an overexpression just in ET-MSC (Fig 5C). Western blot studies of ANGPT1, NF-kB and immunofluorescence assays for CXCL12 were performed. As shown in Fig 6A, MPN-MSC had a higher protein expression of NF-kB whereas almost no expression of ANGPT1 was detected. For CXCL12 expression study (Fig 6B), immunofluorescence showed a decrease in CXCL12 expression in MPN-MSC compared to HD-MSC.

### Cell line co-culture studies

Several lines of evidence suggest that the establishment and progression of MPN can be driven from the crosstalk between the mutated HSPC and the BM microenvironment[5, 31]. In this set of experiments we analyzed if an MPN cell line (UKE-1 and SET-2) with the mutation in JAK2 can change the expression of important key genes, important in the hematopoiesis maintenance, in two different healthy mesenchymal cell lines (hTERT and HS5).

hTERT and HS5 cell lines were maintained in culture with UKE-1, SET2 or CD34^+^ cells (healthy hematopoietic compartment) (*transwell)* for 72h. Then, RNA from mesenchymal cell lines was isolated and the expression of angiopoetin-1, NF-kB and CXCL12 was studied.

**Fig 7:**
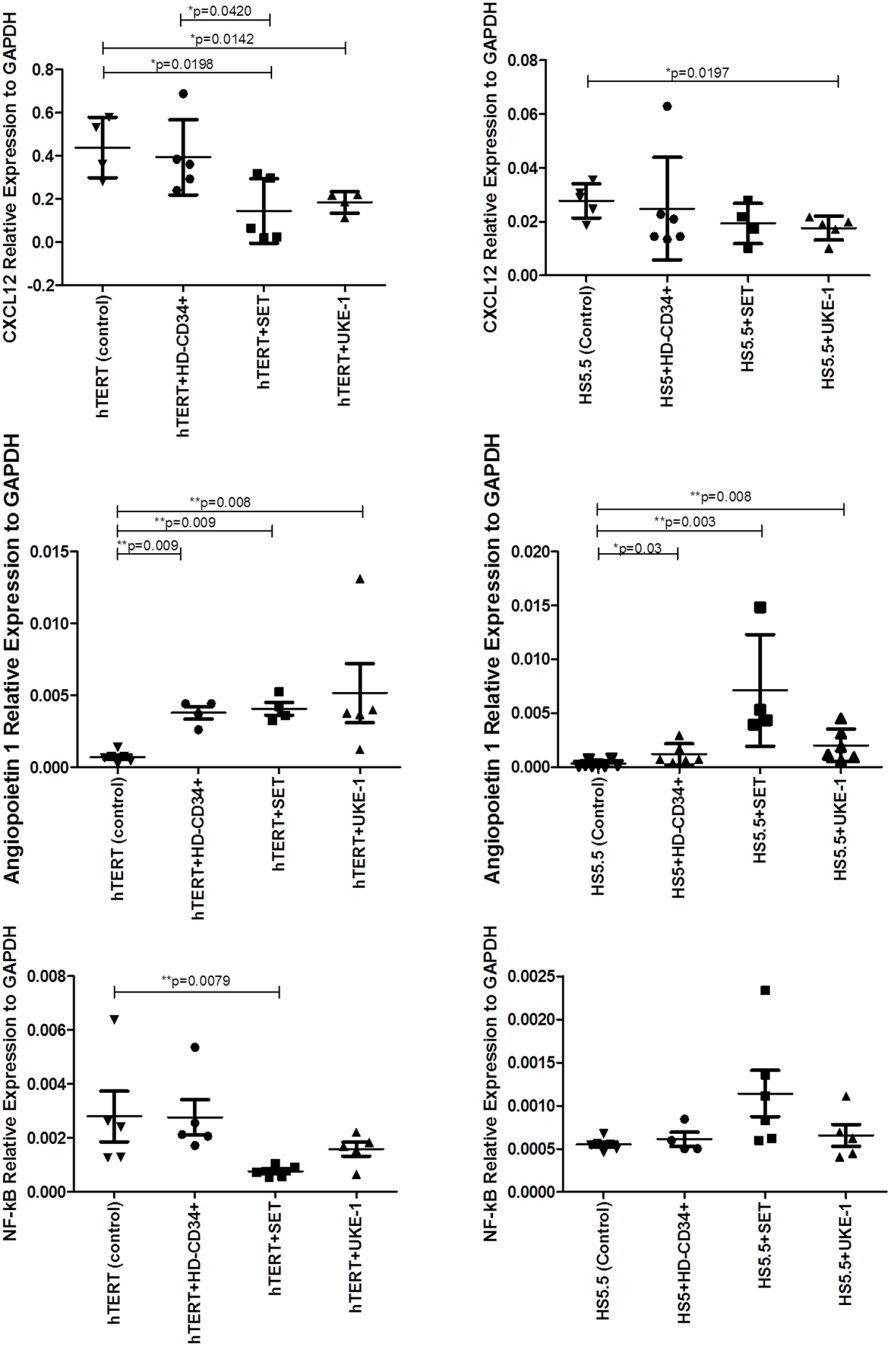
Expression of NF-ƙB and ANGPT-1 and CXCL12 by real-time PCR in MSC cell lines (hTERT and HS5) after co-culture with UKE-1 cells. Results were normalized with the housekeeping gene GAPDH. Results are represented by the median and the interquartile range. (A) Angiopoetin 1. (B) NF-ƙB. (C) CXCL12. The control HS5 and hTERT represents the cells that were co-cultured for 72h with UKE cells (*transwell*) n = 10.

The leukemia-cell- changes in BM-MSC were different that those induced by CD34^+^ cells. Was observed that the culture of leukemic cells (UKE-1 and SET-2) induce changes the expression of CXCL12 in the MSC (healthy). In the case of ANGPT1 expression and a significant increase was observed when both MSC (HS5 and hTERT) were cultured with healthy or neoplastic hematopoietic cells. However, the MSC co-cultured with leukemic cells presented higher expression of this gene (Fig 7). Regarding NF-kB, was observed a decrease in the expression of this gene, when hTERT cells were co-cultured with SET-2 cells.

## Discussion

An increasing number of reports support the fact that mutated HSPC can modify the BM stroma, and these modifications create a positive feedback loop that plays an essential role in MPN progression[5, 31]. Since BM-MSC constitutes a relevant component of the tumor microenvironment in hematological malignancies, in this study we characterize BM-MSC from JAK2V617F patients. The evidence of the concept in which an altered stroma endorsed the clonal HSPC in the development and progression in MPN has been gaining strength[12, 32]. Few and contradictory results of some reports have been described regarding the characterization of BM-derived MSC from negative Ph^-^ MPN patients. Regarding the proliferative and differentiation capabilities of MPN-MSC, we did not observe any glaring differences with HD-MSC. Our results are in accordance with other reports, where the MSC from neoplastic patients do not evidence differences with HD-MSC[12, 33]. However, some studies reported that MSC from MPN patients, mainly from PMF patients, showed slower proliferation rate and reached senescence at earlier passages[11]. Regarding the immunophenotypic characterization of MPN-MSC, MFI expression from some molecules was different, showing an increased MIF for CD73, CD90 and CD44, and decreased of CD105. Numerous studies have previously described discrepancies in the MFI of these markers in the MSC from other hematological disorders[34–37]. Endoglin CD105 is an important molecule in osteoblasts and adipocyte differentiation process[38]. CD44 (hyaluronan receptor) is involved in cell adhesion, migration, and homing of MSC[39]. CD44 activation leads to the stimulation of multiple downstream pathways including TGF-ß signaling in the MSC[40]. The GPI-anchorage proteins, CD73 (ecto-5’ nucleotidase) and CD90 (Thy-1), are signal transduction molecules in the human immune system and mediators of cell-cell and cell-matrix interactions. It has been demonstrated that CD90 knockdown in the MSC facilitates osteogenic and adipogenic differentiation[41]. Exogenous expression of CD90-non-expressing fibroblasts results in RhoGTPase activation[42]. We also study the presence of the mutation JAK2V617F in the MSC from patients, which was negative in our study, as it has been previously reported[10].

Regarding gene expression profiling, our results showed a differential expression pattern in PV and ET-MSC when compared to HD-MSC. Specially, PV-MSC showed the highest number of overexpressed genes. Biological analysis showed that these genes are related to endoplasmic reticulum processing, protein transport and GTPase activity. These signaling pathways are important in cell differentiation, organization and dynamics of actin cytoskeleton, and cell migration[43, 44]. From all overexpressed genes we selected MYADM as an example to confirm our genomic studies, which is a gene involved in the hematopoietic process and is up-regulated during myeloid differentiation[30]. MYADM is a MAL family protein, with eight transmembrane regions located at the plasma membrane. In endothelial cells, MYADM has the capacity to change their migration capacity and control the endothelial inflammatory response[45]. Further studies will be necessary to explain the impact of MYADM overexpression in the MSC from MPN patients. A number of other genes are differentially expressed between normal MSC and MSC from JAK2V617F patients. Their implications and functional validation warrants future experiments. As an example, our group has recently shown the role of the expression of HDAC8 in MSC from these patients [46].

One of the most important functional properties of BM-MSC is their capacity to support hematopoiesis. One of our main objectives in this study was to functionally assess the differences in this hematopoietic-supporting ability between BM-MSC from MPN-patients and normal BM-MSC. Two sets of experiments were performed. Firstly, we analyzed the capacity of BM-MSC to promote clonogenic growth of HSPC. Interestingly, we observed an increase in colony formation when the CD34^+^ cells from JAK2V617F patients were maintained with BM-MSC from MPN patients, providing a competitive advantage for leukemic cells. Results from other groups have suggested that malignant stroma promotes the survival and proliferation of mutated hematopoietic progenitor cells [5, 31]. Secondly, we assessed MPN-MSC capacity to maintain HD-HPC in a long-term culture system. Although we did not observe differences in the total number of CFU-GM produced, the weekly evaluation of the number of colonies generated, showed a significant increase in the number of CFU-GM for the HD-HPC that were co-cultured with JAK2-MSC. Previous studies in this setting have shown contradictory results. There are some reports describing a severely compromised ability to maintain HD-HSPC in MPN-expanded osteoblasts and in human MSC from patients with classical Philadelphia-negative MPN[11, 31]. Conversely, others have indicated that MPN-MSC exhibit similar long-term hematopoiesis support ability compared to HD-MSC[12].

In order to analyze which mechanism could be involved in this increased functional capacity, we analyzed the expression of some genes known to be directly or indirectly involved in hematopoietic support. In MPN, decreased expression of CXCL12 by osteoblasts or by Nestin^+^ cells has been associated with the enhancement of the mobilization and loss of normal HSPC[47, 48]. Immunofluorescence studies showed a decrease in the expression of CXCL12 in the MPN-MSC, showing that our results are in agreement with data from other authors. Other genes related to hematopoietic support were also analyzed. A decrease in the expression of ANGPT1 and an increase of NF-KB, THPO and SPP1 expression in MPN-MSC were observed. ANGPT1 is a gene that regulates angiogenesis, which in the hematopoiesis setting has been associated to the induction of quiescence in HSPC through the interaction with the Tie-2 (CD90) receptor, increasing the adhesion of HSPC to osteoblasts in the BM[49]. Recent studies reported that BM-MSC from leukemia patients express NF-ƙB at higher levels than normal BM-MSC[50]. They also showed that the activation of NF-ƙB signaling can be induced by the leukemic cell, playing a pivotal role in the development of leukemia chemoresistance. Other important HSPC-maintenance transcription factor, THPO, was infra-expressed in ET-MSC. THPO is a very important cytokine in the regulation of megakaryocyte development and platelet production, and it is also involved in the regulation of HSPC survival and proliferation[51]. THPO/ MPL signaling is involved in the HSPC niche regulation, maintaining LT-HSPC quiescence in the osteoblastic niche[52]. In the study reported by Schepers, they identified the role of THPO and CCL3 by which leukemic myeloid cells stimulate MSC to overproduce osteoblasts during BM remodeling in the MPN development[31]. We also observed an increase of SPP1 in the MPN-MSC when compared to HD-MSC, some studies showing alteration in the transcriptome and functional analyses with an increased osteogenic potential and a TGFβ1 signaling signature in the MSC from PMF[12].

Recently, it has been discussed how leukemic hematopoiesis could affect the BM-stroma to create a self-reinforcing leukemic niche that promotes leukemia progression, while negatively affecting normal HSPC function. Our findings, in the present work, show a deregulated expression profile affecting some genes involved in HSPC maintenance. In contrast to what has been reported, a profound deregulation of these genes was found in the MSC from patients with PMF, where the fibrotic state is well established[11, 12, 53]. In our study, the majority of MSC were isolated from BM of MPN at diagnosis, suggesting that during MPN evolution there are genetic alterations involving BM-MSC, which likely may influence disease behavior. It will be interesting to follow up the expression of these genes, and to study if they change during disease progression. Thereafter we established co-culture of hTERT and HS5 with UKE cells, SET cells (MPN cell line) and with CD34^+^ cells (HD), to study the alterations in the expression of important genes (CXCL12, NF-ƘB and Angiopoetin1) induced by leukemic cells. We observed that after 72 hours of culture with MPN cell lines, a significant decrease of CXCL12 expression and an increase of ANGPT1 expression were observed in both MSC cell lines. These results demonstrate the capacity of leukemic cells to interact with stromal cells.

In summary, we have shown that BM-MSC from JAK2V617F patients display different immunophenotype expression, gene expression profile and alterations in the expression of genes related to hematopoietic support. We also observed that MPN-MSC may protect the neoplastic cells by increasing their colony formation capacity. Our results provide new insights to understand the biological role of BM-MSC in the pathophysiology of JAK2V617F MPN.

## Supporting information

S1 Fig Multiparametric flow cytometry immunophenotyping. Left panel (A) shows representative dot-plots of stained BM-MSC from HD and JAK2V617F patients (ET and PV). In the right panel (B) it can be observed scatter dot plot of positive surface marker expression in the BM-MSC from the different groups. (D) Representative histograms of BM-MSC from HD and MPN patients. The line represents the median with interquartile range. MIF–Mean Fluorescence Intensity. (* p˂0.05).
(TIF)

S2 Fig Hematopoietic supporting capacity of MPN-MSC. (A) Graphic shows the total colony-forming unit (CFU) from JAK2V617F-CD34^+^ cells after 48h of culture with PV-MSC and ET-MSC, no differences were observed between groups (B) Capacity of MPN-MSC to maintain HD-HPC in LTBMC. Total of CFU-GM from HD-CD34^+^ cells after 5 weeks in co-culture with PV-MSC (n = 3) and ET-MSC (n = 4).
(TIF)

S1 Table Panel of genes used in RT-PCR assays. (DOCX)

S2 Table Differential Up (red) Down (green)–regulated expression genes in BM-MSC from PV patients (PV-MSC) contrasted against healthy controls (HD-MSC). (DOCX)

S3 Table Differential Up (red) Down (green)–regulated expression genes in BM-MSC from ET patients (ET-MSC) contrasted against healthy controls (HD-MSC). (DOCX)

S4 Table Top-10 genes detected as differentially expressed (Up-regulated) using SAM algorithm. (DOCX)
