# Supplementary figures and images for: Mesenchymal stromal cells (MSC) from JAK2+ myeloproliferative neoplasms differ from normal MSC and contribute to the maintenance of neoplastic hematopoiesis
Source: PLoS One. 2017 Aug 10;12(8):e0182470. doi: 10.1371/journal.pone.0182470 (PMC5552029; doi:10.1371/journal.pone.0182470)

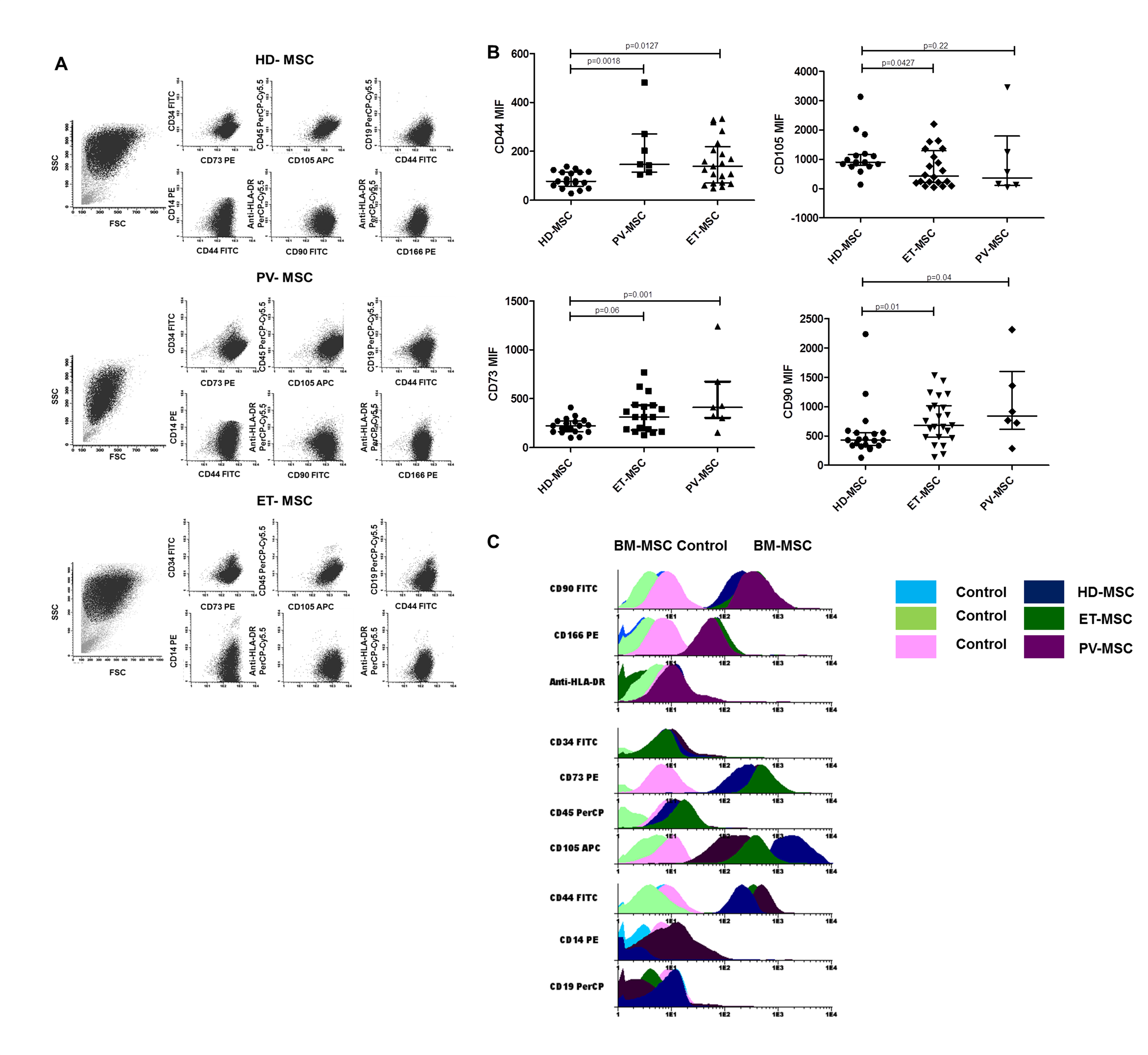

Supplement: S1 Fig — Left panel (A) shows representative dot-plots of stained BM-MSC from HD and JAK2V617F patients (ET and PV). In the right panel (B) it can be observed scatter dot plot of positive surface marker expression in the BM-MSC from the different groups. (D) Representative histograms of BM-MSC from HD and MPN patients. The line represents the median with interquartile range. MIF–Mean Fluorescence Intensity. (* p˂0.05). (TIF) [file pone.0182470.s001.tif]

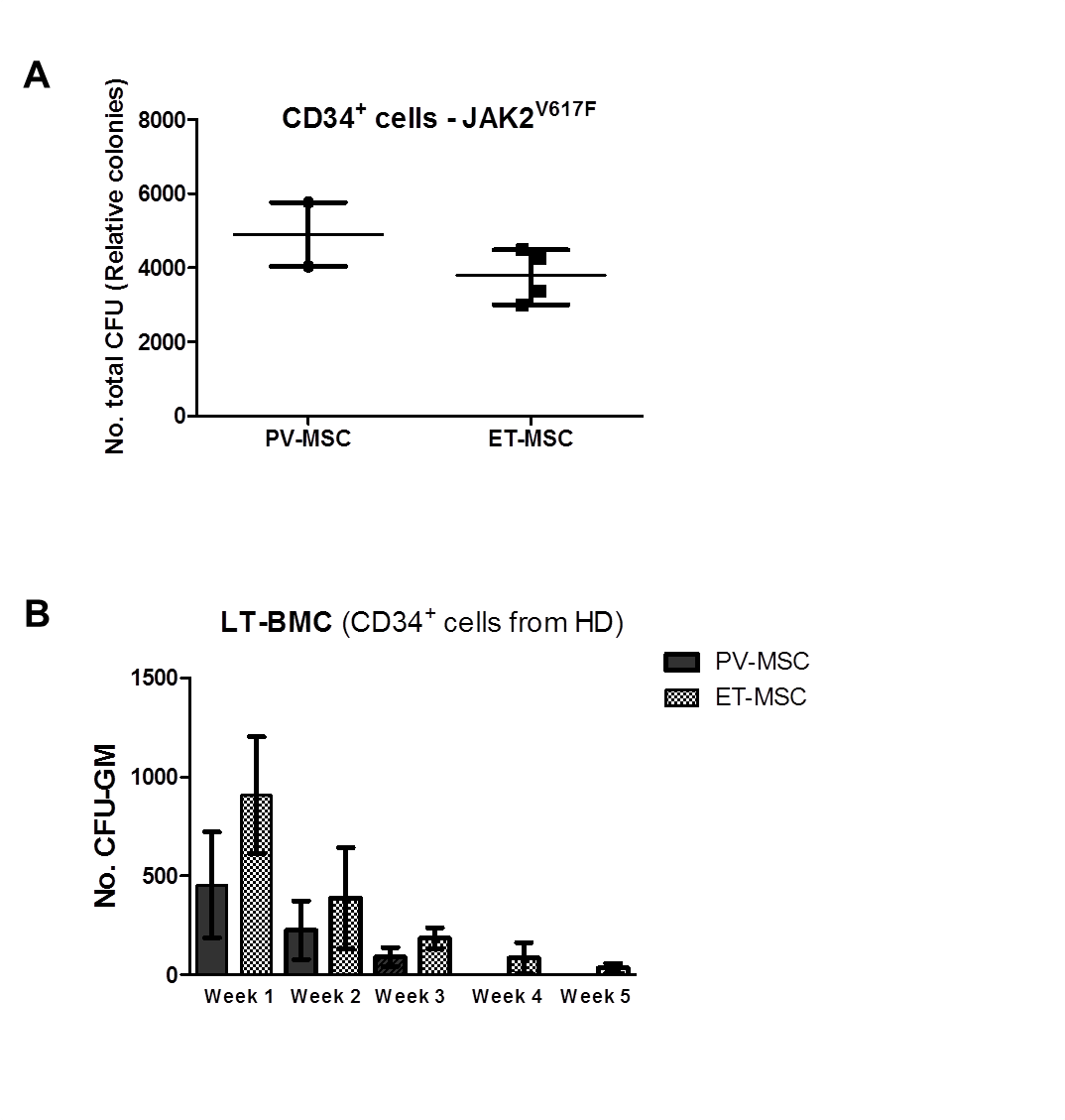

Supplement: S2 Fig — (A) Graphic shows the total colony-forming unit (CFU) from JAK2V617F-CD34+ cells after 48h of culture with PV-MSC and ET-MSC, no differences were observed between groups (B) Capacity of MPN-MSC to maintain HD-HPC in LTBMC. Total of CFU-GM from HD-CD34+ cells after 5 weeks in co-culture with PV-MSC (n = 3) and ET-MSC (n = 4). (TIF) [file pone.0182470.s002.tif]
