# Supplementary material for: Mesenchymal stromal cells (MSC) from JAK2+ myeloproliferative neoplasms differ from normal MSC and contribute to the maintenance of neoplastic hematopoiesis
Source: PLoS One. 2017 Aug 10;12(8):e0182470. doi: 10.1371/journal.pone.0182470 (PMC5552029; doi:10.1371/journal.pone.0182470)
Supplement: S1 Table — (DOCX) [file pone.0182470.s003.docx]

**RNA isolation and gene expression determination**

**S1 Table:** Panel of genes used in RT-PCR assays

| **Gene symbol** | **Assay IDs** |
| --- | --- |
| **KIT** | Hs00174029 |
| **SPP1** | Hs00167093 |
| **THPO** | Hs00171458 |
| **JAG1** | Hs01070032 |
| **BMP2** | Hs01055564 |
| **ANGPT1** | Hs00181613 |
| **NFKBIB** | Hs00182115 |
| **TNF** | Hs00174128 |
| **MYADM** | Hs00414763 |
| **CXCL12** | Hs00171022 |
| **HDAC8** | Hs00954353 |
| **GAPDH** | Hs02758991 |

*All the genes were purchased from Applied Biosystems, Foster City, CA, USA*
