# Supplementary material for: Mesenchymal stromal cells (MSC) from JAK2+ myeloproliferative neoplasms differ from normal MSC and contribute to the maintenance of neoplastic hematopoiesis
Source: PLoS One. 2017 Aug 10;12(8):e0182470. doi: 10.1371/journal.pone.0182470 (PMC5552029; doi:10.1371/journal.pone.0182470)
Supplement: S2 Table — (DOCX) [file pone.0182470.s004.docx]

| **S2 Table: Differential Up (red) Down (green) – regulated expression genes in BM-MSC from PV patients (PV-MSC) contrasted against healthy controls (HD-MSC).** | | | | | | |
| --- | --- | --- | --- | --- | --- | --- |
| **ID** | **logFC** | **AveExpr** | **P.Value** | **adj.P.Val** | **gene_symbol** | **biotype** |
| **ENSG00000179820** | **0,913** | **12,308** | **0,0000** | **0,0003** | **MYADM** | **protein_coding** |
| ENSG00000089693 | 0,88 | 12,1015 | 0,0000 | 0,0077 | MLF2 | protein_coding |
| ENSG00000173914 | 0,619 | 10,2832 | 0,0000 | 0,0077 | RBM4B | protein_coding |
| ENSG00000103005 | 1,03 | 9,10676 | 0,0000 | 0,0097 | C16orf57 | protein_coding |
| ENSG00000115806 | 0,51 | 11,7966 | 0,0000 | 0,0114 | **GORASP2** | **protein_coding** |
| ENSG00000176422 | 0,781 | 8,52383 | 0,0000 | 0,0114 | SPRYD4 | protein_coding |
| ENSG00000101464 | 0,915 | 10,2571 | 0,0000 | 0,0114 | PIGU | protein_coding |
| ENSG00000158825 | 0,871 | 7,14014 | 0,0000 | 0,0114 | **CDA** | **protein_coding** |
| ENSG00000184584 | 1,088 | 9,88173 | 0,0000 | 0,0114 | TMEM173 | protein_coding |
| ENSG00000228834 | 1,041 | 10,6911 | 0,0000 | 0,0114 | RP11-249L21.3 | pseudogene |
| ENSG00000140497 | 0,707 | 11,2176 | 0,0000 | 0,0114 | SCAMP2 | protein_coding |
| ENSG00000100196 | 0,913 | 12,1138 | 0,0000 | 0,0114 | KDELR3 | protein_coding |
| ENSG00000125149 | 0,712 | 9,8079 | 0,0000 | 0,0114 | C16orf70 | protein_coding |
| ENSG00000119414 | 0,551 | 12,2253 | 0,0000 | 0,0114 | PPP6C | protein_coding |
| ENSG00000133678 | 0,719 | 10,9651 | 0,0000 | 0,0114 | RP11-369J21.2 | protein_coding |
| ENSG00000158710 | 0,869 | 11,4756 | 0,0000 | 0,0114 | TAGLN2 | protein_coding |
| ENSG00000086848 | 0,404 | 9,2107 | 0,0000 | 0,0133 | **FDXACB1** | **protein_coding** |
| ENSG00000177981 | 0,475 | 11,1973 | 0,0000 | 0,0146 | **ASB8** | **protein_coding** |
| ENSG00000076067 | 0,867 | 11,5308 | 0,0000 | 0,0146 | RBMS2 | protein_coding |
| ENSG00000105991 | 0,601 | 7,19679 | 0,0000 | 0,0146 | HOXA1 | protein_coding |
| ENSG00000170043 | 0,679 | 12,1044 | 0,0000 | 0,0160 | TRAPPC1 | protein_coding |
| ENSG00000162736 | 0,523 | 11,5362 | 0,0000 | 0,0160 | NCSTN | protein_coding |
| ENSG00000139998 | 1,259 | 7,98604 | 0,0000 | 0,0160 | RAB15 | protein_coding |
| ENSG00000187838 | 0,816 | 10,2464 | 0,0000 | 0,0160 | PLSCR3 | protein_coding |
| ENSG00000147403 | 0,61 | 12,8932 | 0,0000 | 0,0160 | RPL10 | protein_coding |
| ENSG00000134779 | 0,62 | 11,4564 | 0,0000 | 0,0160 | C18orf10 | protein_coding |
| ENSG00000141756 | 0,477 | 12,6345 | 0,0000 | 0,0160 | FKBP10 | protein_coding |
| ENSG00000147099 | 0,598 | 9,87112 | 0,0000 | 0,0160 | HDAC8 | protein_coding |
| ENSG00000120509 | 0,639 | 8,17884 | 0,0000 | 0,0166 | PDZD11 | protein_coding |
| ENSG00000144043 | 0,653 | 11,2335 | 0,0000 | 0,0180 | **TEX261** | **protein_coding** |
| ENSG00000103429 | 0,505 | 10,7062 | 0,0000 | 0,0180 | BFAR | protein_coding |
| ENSG00000180304 | 0,773 | 9,58422 | 0,0000 | 0,0190 | OAZ2 | protein_coding |
| ENSG00000188917 | 0,546 | 8,11408 | 0,0000 | 0,0196 | TRMT2B | protein_coding |
| ENSG00000134287 | 0,564 | 12,0757 | 0,0000 | 0,0201 | ARF3 | protein_coding |
| ENSG00000124164 | 0,426 | 9,98444 | 0,0000 | 0,0201 | **VAPB** | **protein_coding** |
| ENSG00000143252 | 0,716 | 8,91027 | 0,0000 | 0,0203 | SDHC | protein_coding |
| ENSG00000102119 | 0,934 | 10,6576 | 0,0000 | 0,0224 | EMD | protein_coding |
| ENSG00000158161 | 0,646 | 8,72548 | 0,0000 | 0,0224 | EYA3 | protein_coding |
| ENSG00000168227 | 0,306 | 4,99745 | 0,0000 | 0,0227 | AL079307.1 | pseudogene |
| ENSG00000011198 | 0,678 | 10,3454 | 0,0000 | 0,0227 | ABHD5 | protein_coding |
| ENSG00000213760 | 1,151 | 7,79946 | 0,0000 | 0,0227 | ATP6V1G2 | protein_coding |
| ENSG00000087111 | 0,727 | 10,4043 | 0,0000 | 0,0227 | PIGS | protein_coding |
| ENSG00000159063 | 0,511 | 10,9635 | 0,0000 | 0,0227 | ALG8 | protein_coding |
| ENSG00000025800 | 0,419 | 11,359 | 0,0000 | 0,0227 | KPNA6 | protein_coding |
| ENSG00000214160 | 0,773 | 11,2461 | 0,0001 | 0,0245 | ALG3 | protein_coding |
| ENSG00000173402 | 0,736 | 10,3811 | 0,0001 | 0,0246 | DAG1 | protein_coding |
| ENSG00000131779 | 0,78 | 10,7443 | 0,0001 | 0,0246 | PEX11B | protein_coding |
| ENSG00000134910 | 0,356 | 12,7155 | 0,0001 | 0,0246 | STT3A | protein_coding |
| ENSG00000206697 | 0,247 | 4,70656 | 0,0001 | 0,0246 | Y_RNA | misc_RNA |
| ENSG00000127463 | 0,502 | 10,1881 | 0,0001 | 0,0246 | KIAA0090 | protein_coding |
| ENSG00000109066 | 0,951 | 9,28432 | 0,0001 | 0,0246 | TMEM104 | protein_coding |
| ENSG00000144231 | 0,535 | 9,86265 | 0,0001 | 0,0246 | POLR2D | protein_coding |
| ENSG00000159840 | 0,738 | 12,0045 | 0,0001 | 0,0246 | ZYX | protein_coding |
| ENSG00000177105 | 0,705 | 10,8891 | 0,0001 | 0,0246 | RHOG | protein_coding |
| ENSG00000171792 | 0,664 | 10,4625 | 0,0001 | 0,0246 | C12orf32 | protein_coding |
| ENSG00000100865 | 0,548 | 10,0112 | 0,0001 | 0,0246 | CINP | protein_coding |
| ENSG00000105341 | 0,552 | 9,89154 | 0,0001 | 0,0246 | ATP5SL | protein_coding |
| ENSG00000126970 | 0,71 | 8,36453 | 0,0001 | 0,0246 | ZC4H2 | protein_coding |
| ENSG00000214517 | 0,844 | 11,5372 | 0,0001 | 0,0246 | PPME1 | protein_coding |
| ENSG00000176871 | 0,652 | 11,7434 | 0,0001 | 0,0246 | WSB2 | protein_coding |
| ENSG00000173065 | 0,614 | 9,15411 | 0,0001 | 0,0250 | C17orf63 | protein_coding |
| ENSG00000108518 | 0,651 | 12,5405 | 0,0001 | 0,0274 | PFN1 | protein_coding |
| ENSG00000237758 | 0,783 | 11,2674 | 0,0001 | 0,0274 | AC084031.2 | pseudogene |
| ENSG00000109519 | 0,529 | 9,76648 | 0,0001 | 0,0274 | GRPEL1 | protein_coding |
| ENSG00000184988 | 1,278 | 8,74942 | 0,0001 | 0,0283 | TMEM106A | protein_coding |
| ENSG00000177370 | 0,558 | 10,6932 | 0,0001 | 0,0284 | TIMM22 | protein_coding |
| ENSG00000122203 | 0,666 | 11,0962 | 0,0001 | 0,0286 | KIAA1191 | protein_coding |
| ENSG00000103121 | 0,617 | 11,7125 | 0,0001 | 0,0301 | C16orf61 | protein_coding |
| ENSG00000139579 | 0,65 | 10,74 | 0,0001 | 0,0305 | OBFC2B | protein_coding |
| ENSG00000126067 | 0,419 | 11,9904 | 0,0001 | 0,0305 | PSMB2 | protein_coding |
| ENSG00000183444 | 0,975 | 9,90172 | 0,0001 | 0,0305 | AC004967.6 | pseudogene |
| ENSG00000213533 | 0,706 | 10,0429 | 0,0001 | 0,0310 | TMEM110 | protein_coding |
| ENSG00000102007 | 0,772 | 12,4461 | 0,0001 | 0,0310 | PLP2 | protein_coding |
| ENSG00000131467 | 0,583 | 11,4473 | 0,0001 | 0,0316 | PSME3 | protein_coding |
| ENSG00000187446 | 0,527 | 12,3693 | 0,0001 | 0,0316 | AC012652.1 | protein_coding |
| ENSG00000163191 | 0,538 | 13,0987 | 0,0001 | 0,0328 | S100A11 | protein_coding |
| ENSG00000171889 | 1,383 | 9,35891 | 0,0001 | 0,0333 | RP11+354P17.1 | processed_transcript |
| ENSG00000130731 | 0,611 | 8,98163 | 0,0001 | 0,0335 | C16orf13 | protein_coding |
| ENSG00000176340 | 0,926 | 11,1231 | 0,0001 | 0,0349 | COX8A | protein_coding |
| ENSG00000140830 | 0,605 | 8,6955 | 0,0001 | 0,0355 | TXNL4B | protein_coding |
| ENSG00000174903 | 0,745 | 12,1389 | 0,0001 | 0,0355 | RAB1B | protein_coding |
| ENSG00000160746 | 0,682 | 10,8961 | 0,0001 | 0,0355 | ANO10 | protein_coding |
| ENSG00000147100 | 1,411 | 9,59391 | 0,0001 | 0,0355 | SLC16A2 | protein_coding |
| ENSG00000147224 | 1,437 | 10,5916 | 0,0001 | 0,0355 | PRPS1 | protein_coding |
| ENSG00000219200 | 0,448 | 13,2483 | 0,0001 | 0,0355 | RNASEK | protein_coding |
| ENSG00000050438 | 0,648 | 6,32168 | 0,0001 | 0,0355 | SLC4A8 | protein_coding |
| ENSG00000106511 | 0,984 | 8,48998 | 0,0001 | 0,0355 | MEOX2 | protein_coding |
| ENSG00000221988 | 0,932 | 7,7227 | 0,0001 | 0,0362 | PPT2 | protein_coding |
| ENSG00000172992 | 0,904 | 11,0507 | 0,0001 | 0,0362 | DCAKD | protein_coding |
| ENSG00000109062 | 0,682 | 9,35106 | 0,0001 | 0,0362 | SLC9A3R1 | protein_coding |
| ENSG00000114395 | 0,799 | 8,19884 | 0,0001 | 0,0362 | CYB561D2 | protein_coding |
| ENSG00000127329 | 0,64 | 5,85214 | 0,0002 | 0,0362 | PTPRB | protein_coding |
| ENSG00000172757 | 0,531 | 13,0548 | 0,0002 | 0,0362 | CFL1 | protein_coding |
| ENSG00000141030 | 0,413 | 10,7665 | 0,0002 | 0,0362 | COPS3 | protein_coding |
| ENSG00000108774 | 0,382 | 12,9279 | 0,0002 | 0,0362 | RAB5C | protein_coding |
| ENSG00000101337 | 0,497 | 10,8953 | 0,0002 | 0,0371 | TM9SF4 | protein_coding |
| ENSG00000160446 | 0,7 | 10,4786 | 0,0002 | 0,0371 | ZDHHC12 | protein_coding |
| ENSG00000136240 | 0,422 | 12,5765 | 0,0002 | 0,0375 | KDELR2 | protein_coding |
| ENSG00000141499 | 0,463 | 7,89447 | 0,0002 | 0,0381 | WRAP53 | protein_coding |
| ENSG00000023902 | 0,778 | 9,27268 | 0,0002 | 0,0381 | PLEKHO1 | protein_coding |
| ENSG00000111669 | 0,582 | 10,4189 | 0,0002 | 0,0381 | TPI1 | protein_coding |
| ENSG00000243279 | 1,024 | 11,0704 | 0,0002 | 0,0387 | PRAF2 | protein_coding |
| ENSG00000241468 | 0,83 | 10,9631 | 0,0002 | 0,0387 | ATP5J2 | protein_coding |
| ENSG00000135956 | 0,635 | 11,4194 | 0,0002 | 0,0387 | TMEM127 | protein_coding |
| ENSG00000162419 | 0,48 | 8,96449 | 0,0002 | 0,0399 | GMEB1 | protein_coding |
| ENSG00000139531 | 0,618 | 8,13392 | 0,0002 | 0,0407 | SUOX | protein_coding |
| ENSG00000160714 | 0,379 | 11,4609 | 0,0002 | 0,0417 | UBE2Q1 | protein_coding |
| ENSG00000120708 | 1,129 | 12,7278 | 0,0002 | 0,0420 | TGFBI | protein_coding |
| ENSG00000157216 | 0,528 | 10,1115 | 0,0002 | 0,0420 | SSBP3 | protein_coding |
| ENSG00000187534 | 0,851 | 10,5616 | 0,0002 | 0,0422 | AC007842.1 | pseudogene |
| ENSG00000125971 | 0,43 | 10,4283 | 0,0002 | 0,0423 | DYNLRB1 | protein_coding |
| ENSG00000105438 | 0,573 | 11,8186 | 0,0002 | 0,0425 | KDELR1 | protein_coding |
| ENSG00000170633 | 0,48 | 9,2245 | 0,0002 | 0,0425 | RNF34 | protein_coding |
| ENSG00000144120 | 0,582 | 8,25572 | 0,0002 | 0,0425 | TMEM177 | protein_coding |
| ENSG00000197728 | 1,21 | 9,39335 | 0,0002 | 0,0425 | RPS26 | protein_coding |
| ENSG00000062485 | 0,523 | 11,6361 | 0,0002 | 0,0425 | CS | protein_coding |
| ENSG00000128340 | 1,56 | 9,07757 | 0,0002 | 0,0425 | RAC2 | protein_coding |
| ENSG00000135074 | 1,409 | 8,65956 | 0,0002 | 0,0425 | ADAM19 | protein_coding |
| ENSG00000228285 | 0,702 | 8,81161 | 0,0002 | 0,0430 | LYPLA2P1 | pseudogene |
| ENSG00000169372 | 0,485 | 8,62715 | 0,0002 | 0,0430 | **CRADD** | **protein_coding** |
| ENSG00000123353 | 0,597 | 11,0127 | 0,0002 | 0,0435 | ORMDL2 | protein_coding |
| ENSG00000155366 | 0,654 | 12,183 | 0,0002 | 0,0435 | RHOC | protein_coding |
| ENSG00000132801 | 0,542 | 6,9645 | 0,0002 | 0,0438 | ZSWIM3 | protein_coding |
| ENSG00000141349 | 0,742 | 10,8263 | 0,0002 | 0,0445 | G6PC3 | protein_coding |
| ENSG00000127780 | 0,215 | 4,88522 | 0,0002 | 0,0445 | OR1E2 | protein_coding |
| ENSG00000142230 | 0,505 | 10,7459 | 0,0003 | 0,0453 | SAE1 | protein_coding |
| ENSG00000239334 | 0,36 | 5,61432 | 0,0003 | 0,0453 | GSTTP2 | processed_transcript |
| ENSG00000136715 | 0,595 | 10,4446 | 0,0003 | 0,0453 | SAP130 | protein_coding |
| ENSG00000109079 | 0,721 | 10,8449 | 0,0003 | 0,0453 | TNFAIP1 | protein_coding |
| ENSG00000137073 | 0,469 | 10,1549 | 0,0003 | 0,0467 | UBAP2 | protein_coding |
| ENSG00000092847 | 0,399 | 10,0216 | 0,0003 | 0,0467 | EIF2C1 | protein_coding |
| ENSG00000182195 | 1,059 | 10,0649 | 0,0003 | 0,0467 | LDOC1 | protein_coding |
| ENSG00000100558 | 0,74 | 6,59212 | 0,0003 | 0,0469 | PLEK2 | protein_coding |
| ENSG00000231192 | 0,221 | 4,32163 | 0,0003 | 0,0475 | OR5H1 | protein_coding |
| ENSG00000174021 | 0,559 | 10,8632 | 0,0003 | 0,0475 | GNG5 | protein_coding |
| ENSG00000077721 | 0,646 | 11,0012 | 0,0003 | 0,0475 | UBE2A | protein_coding |
| ENSG00000119777 | 0,536 | 11,4247 | 0,0003 | 0,0476 | TMEM214 | protein_coding |
| ENSG00000151348 | 0,332 | 11,846 | 0,0003 | 0,0476 | EXT2 | protein_coding |
| ENSG00000118680 | 0,333 | 13,0533 | 0,0003 | 0,0490 | MYL12B | protein_coding |
| ENSG00000159335 | 0,625 | 11,2267 | 0,0003 | 0,0490 | PTMS | protein_coding |
| ENSG00000081177 | 0,279 | 9,43746 | 0,0003 | 0,0499 | EXD2 | protein_coding |
| ENSG00000138738 | 0,645 | 8,29697 | 0,0003 | 0,0467 | PRDM5 | protein_coding |
| ENSG00000199438 | 0,457 | 6,28652 | 0,0003 | 0,0466 | 5S_rRNA | rRNA |
| ENSG00000116117 | 0,725 | 8,80956 | 0,0003 | 0,0451 | PARD3B | protein_coding |
| ENSG00000065320 | 0,393 | 8,13004 | 0,0002 | 0,0423 | NTN1 | protein_coding |
| ENSG00000125691 | 0,422 | 12,3995 | 0,0002 | 0,0391 | RPL23 | protein_coding |
| ENSG00000148296 | 0,347 | 7,2579 | 0,0002 | 0,0375 | SURF6 | protein_coding |
| ENSG00000242900 | 1,257 | 6,16595 | 0,0001 | 0,0314 | AC097526.11 | Mt_tRNA_pseudogene |
| ENSG00000100650 | 0,775 | 10,7951 | 0,0001 | 0,0305 | SFRS5 | protein_coding |
| ENSG00000154114 | 0,537 | 10,1584 | 0,0001 | 0,0305 | TBCEL | protein_coding |
| ENSG00000188375 | 0,552 | 11,1899 | 0,0001 | 0,0274 | H3F3C | protein_coding |
| ENSG00000244577 | 0,416 | 8,56961 | 0,0000 | 0,0227 | AC137932.3 | scRNA_pseudogene |
| ENSG00000242752 | 1,652 | 5,99731 | 0,0000 | 0,0192 | AC025627.1 | Mt_tRNA_pseudogene |
| ENSG00000169871 | 0,471 | 8,17502 | 0,0000 | 0,0160 | TRIM56 | protein_coding |
| ENSG00000240234 | 1,952 | 8,53154 | 0,0000 | 0,0114 | AC131055.17 | Mt_tRNA_pseudogene |
| ENSG00000242240 | 2,152 | 9,57558 | 0,0000 | 0,0114 | AC073869.19 | Mt_tRNA_pseudogene |
| ENSG00000153046 | 0,515 | 8,95551 | 0,0000 | 0,0077 | CDYL | protein_coding |
