# Supplementary material for: Mesenchymal stromal cells (MSC) from JAK2+ myeloproliferative neoplasms differ from normal MSC and contribute to the maintenance of neoplastic hematopoiesis
Source: PLoS One. 2017 Aug 10;12(8):e0182470. doi: 10.1371/journal.pone.0182470 (PMC5552029; doi:10.1371/journal.pone.0182470)
Supplement: S3 Table — (DOCX) [file pone.0182470.s005.docx]

| **S3 Table: Differential Up (red) Down (green) – regulated expression genes in BM-MSC from ET patients (ET-MSC) contrasted against healthy controls (HD-MSC).** | | | | | | |
| --- | --- | --- | --- | --- | --- | --- |
| **ID** | **logFC** | **AveExpr** | **P.Value** | **adj.P.Val** | **gene_symbol** | **biotype** |
| ENSG00000127946 | 0,972 | 8,408285 | 0,0000 | 0,0257 | HIP1 | protein_coding |
| ENSG00000228834 | 0,906 | 10,69114 | 0,0000 | 0,0315 | RP11-249L21.3 | pseudogene |
| ENSG00000243685 | 0,833 | 5,752575 | 0,0000 | 0,0026 | AL122001.1 | scRNA_pseudogene |
| ENSG00000101464 | 0,814 | 10,25713 | 0,0000 | 0,0257 | **PIGU** | **protein_coding** |
| ENSG00000143252 | 0,772 | 8,910272 | 0,0000 | 0,0257 | SDHC | protein_coding |
| ENSG00000133678 | 0,76 | 10,96507 | 0,0000 | 0,0185 | **TMEM254** | **protein_coding** |
| **ENSG00000179820** | **0,744** | **12,30798** | **0,0000** | **0,0041** | **MYADM** | **protein_coding** |
| ENSG00000119242 | 0,724 | 11,01909 | 0,0001 | 0,0467 | CCDC92 | protein_coding |
| ENSG00000089693 | 0,714 | 12,10153 | 0,0000 | 0,0257 | **MLF2** | **protein_coding** |
| ENSG00000176422 | 0,682 | 8,523831 | 0,0000 | 0,0257 | **SPRYD4** | **protein_coding** |
| ENSG00000173914 | 0,609 | 10,28316 | 0,0000 | 0,0124 | **RBM4B** | **protein_coding** |
| ENSG00000140497 | 0,601 | 11,21762 | 0,0000 | 0,0394 | SCAMP2 | protein_coding |
| ENSG00000187446 | 0,591 | 12,36932 | 0,0000 | 0,0266 | AC012652.1 | protein_coding |
| ENSG00000162419 | 0,578 | 8,964494 | 0,0000 | 0,0257 | GMEB1 | protein_coding |
| ENSG00000134779 | 0,566 | 11,4564 | 0,0001 | 0,0463 | C18orf10 | protein_coding |
| ENSG00000081853 | 0,557 | 8,182415 | 0,0000 | 0,0257 | **PCDHGC5** | **protein_coding** |
| ENSG00000119414 | 0,532 | 12,22531 | 0,0000 | 0,0257 | PPP6C | protein_coding |
| ENSG00000177981 | 0,513 | 11,19727 | 0,0000 | 0,0185 | ASB8 | protein_coding |
| ENSG00000214770 | 0,457 | 5,461314 | 0,0000 | 0,0256 | AL161756.1 | protein_coding |
| ENSG00000141030 | 0,457 | 10,76647 | 0,0000 | 0,0391 | **COPS3** | **protein_coding** |
| ENSG00000115806 | 0,456 | 11,79658 | 0,0000 | 0,0257 | GORASP2 | protein_coding |
| ENSG00000165078 | 0,429 | 5,3891 | 0,0000 | 0,0256 | CPA6 | protein_coding |
| ENSG00000201957 | 0,201 | 4,51223 | 0,0000 | 0,0257 | SNORA25 | snoRNA |
| ENSG00000172487 | 0,197 | 4,686754 | 0,0001 | 0,0488 | OR8J1 | protein_coding |
| ENSG00000243202 | 0,3146 | 5,577353 | 0,0000 | 0,0266 | AC107956.1 | scRNA_pseudogene |
| ENSG00000244112 | 0,3729 | 6,051949 | 0,0000 | 0,0266 | AC087885.1 | scRNA_pseudogene |
| ENSG00000188732 | 0,3816 | 5,597397 | 0,0001 | 0,0467 | C7orf46 | protein_coding |
| ENSG00000153046 | 0,4092 | 8,955514 | 0,0000 | 0,0266 | CDYL | protein_coding |
| ENSG00000120853 | 0,4245 | 6,254706 | 0,0000 | 0,0257 | GOLGA2L1 | protein_coding |
| ENSG00000169871 | 0,4251 | 8,175019 | 0,0001 | 0,0467 | TRIM56 | protein_coding |
| ENSG00000148296 | 0,4255 | 7,257902 | 0,0000 | 0,0257 | SURF6 | protein_coding |
| ENSG00000244577 | 0,4482 | 8,569607 | 0,0000 | 0,0257 | AC137932.3 | scRNA_pseudogene |
| ENSG00000125691 | 0,5036 | 12,39951 | 0,0000 | 0,0257 | RPL23 | protein_coding |
| ENSG00000242900 | 1,3902 | 6,165952 | 0,0000 | 0,0278 | AC097526.11 | Mt_tRNA_pseudogene |
| ENSG00000240234 | 1,8453 | 8,531542 | 0,0000 | 0,0257 | AC131055.17 | Mt_tRNA_pseudogene |
| ENSG00000242240 | 2,0915 | 9,57558 | 0,0000 | 0,0185 | AC073869.19 | Mt_tRNA_pseudogene |
