# Supplementary material for: Mesenchymal stromal cells (MSC) from JAK2+ myeloproliferative neoplasms differ from normal MSC and contribute to the maintenance of neoplastic hematopoiesis
Source: PLoS One. 2017 Aug 10;12(8):e0182470. doi: 10.1371/journal.pone.0182470 (PMC5552029; doi:10.1371/journal.pone.0182470)
Supplement: S4 Table — (DOCX) [file pone.0182470.s006.docx]

**S4 Table:** **Top-10 genes detected as differentially expressed (Up-regulated) using SAM algorithm** for the contrast of BM-MSC from ET patients versus healthy controls (4 ET-MSC versus 10 HD-MSC) and for the contrast of BM-MSC from PV patients versus healthy controls (4 PV-MSC versus 10 HD-MSC). In blue and bold are the genes that were also found Up-regulated in the differential expression analyses done with LIMMA algorithm (8 genes out of the top-10 from SAM were found by the two methods in their respective significant lists).

| **gene_ID_ENSG** | **gene_symbol** | **contrast** | **d.value** | **p.value** | **q.value** | **logFC** | **biotype** | **gene_description** |
| --- | --- | --- | --- | --- | --- | --- | --- | --- |
| ENSG00000133678 | **TMEM254** | 4ETv10HD | 9.662 | 0.0000012 | **0.0228456** | 1.693 | protein_coding | Transmembrane protein C10orf57 [UniProt::Q8TBM7] |
| ENSG00000081853 | **PCDHGC5** | 4ETv10HD | 7.271 | 0.0000148 | **0.0581524** | 1.471 | protein_coding | protocadherin gamma subfamily C, 5 [HGNC:8718] |
| ENSG00000179820 | **MYADM** | 4ETv10HD | 7.147 | 0.0000153 | **0.0581524** | **1.675** | protein_coding | myeloid-associated differentiation marker [HGNC:7544] |
| ENSG00000006638 | TBXA2R | 4ETv10HD | 6.745 | 0.0000257 | 0.0581524 | 1.562 | protein_coding | thromboxane A2 receptor [HGNC:11608] |
| ENSG00000089693 | **MLF2** | 4ETv10HD | 6.672 | 0.0000273 | **0.0581524** | 1.641 | protein_coding | myeloid leukemia factor 2 [HGNC:7126] |
| ENSG00000173914 | **RBM4B** | 4ETv10HD | 6.658 | 0.0000273 | **0.0581524** | 1.525 | protein_coding | RNA binding motif protein 4B [HGNC:28842] |
| ENSG00000176422 | **SPRYD4** | 4ETv10HD | 6.626 | 0.0000293 | **0.0581524** | 1.604 | protein_coding | SPRY domain containing 4 [HGNC:27468] |
| ENSG00000101464 | **PIGU** | 4ETv10HD | 6.577 | 0.0000329 | **0.0581524** | 1.758 | protein_coding | phosphatidylinositol glycan anchor biosynthesis, class U [HGNC:15791] |
| ENSG00000141030 | **COPS3** | 4ETv10HD | 6.561 | 0.0000337 | **0.0581524** | 1.372 | protein_coding | COP9 constitutive photomorphogenic homolog subunit 3 [HGNC:2239] |
| ENSG00000108272 | DHRS11 | 4ETv10HD | 6.403 | 0.0000441 | 0.0698060 | 1.492 | protein_coding | dehydrogenase/reductase (SDR family) member 11 [HGNC:28639] |
| ENSG00000185873 | TMPRSS11B | 4PVv10HD | 9.812 | 0.0000040 | 0.0354580 | 1.117 | protein_coding | transmembrane protease, serine 11B [HGNC:25398] |
| ENSG00000124164 | **VAPB** | 4PVv10HD | 9.262 | 0.0000080 | **0.0472774** | 1.343 | protein_coding | (vesicle-associated membrane protein)-associated protein B and C [HGNC:12649] |
| ENSG00000177981 | **ASB8** | 4PVv10HD | 8.538 | 0.0000136 | **0.0602786** | 1.390 | protein_coding | ankyrin repeat and SOCS box-containing 8 [HGNC:17183] |
| ENSG00000179820 | **MYADM** | 4PVv10HD | 8.311 | 0.0000181 | **0.0638244** | **1.883** | protein_coding | myeloid-associated differentiation marker [HGNC:7544] |
| ENSG00000169372 | **CRADD** | 4PVv10HD | 7.991 | 0.0000281 | **0.0827354** | 1.400 | protein_coding | CASP2 and RIPK1 domain containing adaptor with death domain [HGNC:2340] |
| ENSG00000172487 | OR8J1 | 4PVv10HD | 7.665 | 0.0000450 | 0.0847356 | 1.117 | protein_coding | olfactory receptor, family 8, subfamily J, member 1 [HGNC:14855] |
| ENSG00000115806 | **GORASP2** | 4PVv10HD | 7.451 | 0.0000594 | **0.0847356** | 1.424 | protein_coding | golgi reassembly stacking protein 2, 55kDa [HGNC:17500] |
| ENSG00000158825 | **CDA** | 4PVv10HD | 7.444 | 0.0000610 | **0.0847356** | 1.829 | protein_coding | cytidine deaminase [HGNC:1712] |
| ENSG00000086848 | **FDXACB1** | 4PVv10HD | 7.184 | 0.0000815 | **0.0847356** | 1.323 | protein_coding | asparagine-linked glycosylation 9, alpha-1,2-mannosyltransferase [HGNC:15672] |
| ENSG00000144043 | **TEX261** | 4PVv10HD | 7.169 | 0.0000827 | **0.0847356** | 1.573 | protein_coding | testis expressed 261 [HGNC:30712] |
